# Natural variation in LONELY GUY-Like 1 regulates rice grain weight under warmer night conditions

**DOI:** 10.1093/plphys/kiae313

**Published:** 2024-05-31

**Authors:** Jaspreet Sandhu, Larissa Irvin, Anil Kumar Chandaran, Shohei Oguro, Puneet Paul, Balpreet Dhatt, Waseem Hussain, Shannon S Cunningham, Cherryl O Quinones, Argelia Lorence, Maria Arlene Adviento-Borbe, Paul Staswick, Gota Morota, Harkamal Walia

**Affiliations:** Department of Agronomy and Horticulture, University of Nebraska-Lincoln, Lincoln, NE 68583, USA; Department of Agronomy and Horticulture, University of Nebraska-Lincoln, Lincoln, NE 68583, USA; Department of Agronomy and Horticulture, University of Nebraska-Lincoln, Lincoln, NE 68583, USA; Department of Agronomy and Horticulture, University of Nebraska-Lincoln, Lincoln, NE 68583, USA; Department of Agronomy and Horticulture, University of Nebraska-Lincoln, Lincoln, NE 68583, USA; Department of Agronomy and Horticulture, University of Nebraska-Lincoln, Lincoln, NE 68583, USA; Department of Agronomy and Horticulture, University of Nebraska-Lincoln, Lincoln, NE 68583, USA; International Rice Research Institute (IRRI), Los Baños, Laguna 4031, Philippines; Department of Chemistry and Physics, Arkansas Biosciences Institute, Arkansas State University, Jonesboro, AR 72467, USA; Department of Chemistry and Physics, Arkansas Biosciences Institute, Arkansas State University, Jonesboro, AR 72467, USA; Department of Chemistry and Physics, Arkansas Biosciences Institute, Arkansas State University, Jonesboro, AR 72467, USA; Delta Water Management Research Unit, USDA-ARS, Jonesboro, AR 72401, USA; Department of Agronomy and Horticulture, University of Nebraska-Lincoln, Lincoln, NE 68583, USA; Department of Animal and Poultry Sciences, Virginia Polytechnic Institute and State University, Blacksburg, VA 24061, USA; Department of Agronomy and Horticulture, University of Nebraska-Lincoln, Lincoln, NE 68583, USA

## Abstract

Global nighttime temperatures are rising at twice the rate of daytime temperatures and pose a challenge for rice (*Oryza sativa*) production. High nighttime temperature (HNT) stress affects rice yield by reducing grain weight, size, and fertility. Although the genes associated with these yield parameters have been identified and characterized under normal temperatures, the genetic basis of grain weight regulation under HNT stress remains less explored. We examined the natural variation for rice single grain weight (SGW) under HNT stress imposed during grain development. A genome-wide association analysis identified several loci associated with grain weight under HNT stress. A locus, *SGW1*, specific to HNT conditions resolved to *LONELY GUY-Like 1* (*LOGL1*), which encodes a putative cytokinin-activation enzyme. We demonstrated that *LOGL1* contributes to allelic variation at *SGW1*. Accessions with lower *LOGL1* transcript abundance had higher grain weight under HNT. This was supported by the higher grain weight of *logl1*-mutants relative to the wild type under HNT. Compared to *logl1*-mutants, *LOGL1* over-expressers showed increased sensitivity to HNT. We showed that LOGL1 regulates the thiamin biosynthesis pathway, which is under circadian regulation, which in turn is likely perturbed by HNT stress. These findings provide a genetic source to enhance rice adaptation to warming night temperatures and improve our mechanistic understanding of HNT stress tolerance pathways.

## Teaser

Allelic variation in *LOGL1* regulates grain weight under high night temperature during reproductive development in rice.

## Introduction

The global average temperatures are rising more rapidly than in the previous century (IPCC 2018). This has been shown to negatively impact the yields of major food crops such as wheat (*Triticum aestivum*), rice (*Oryza sativa*), and maize (*Zea mays*) (Peng et al. 2004; Wheeler and Von Braun 2013; Asseng et al. 2015; Zhao et al. 2017). Notably, the daily minimum (nighttime) temperatures are increasing more rapidly than daily maximum (daytime) temperatures, thus decreasing the diurnal temperature range (Easterling et al. 1997; Vose et al. 2005; Welch et al. 2010; Wang et al. 2017). This diurnal asymmetry in warming may have unique consequences on crop yield, likely due to differential impact on daytime carbon assimilation (via photosynthesis) and nighttime carbon consumption (via respiration) (Donat and Alexander 2012; Peng et al. 2013; Xia et al. 2014; Wang et al. 2017). Hence, high-yielding cultivars, which have been developed under optimal diurnal temperature range, can become more sensitive to diurnal asymmetry caused by high nighttime temperature (HNT) (Jagadish et al. 2015). Rice is a staple crop for many developing countries that are more likely to experience warmer nights (Vose et al. 2005; Cox et al. 2020). Field studies indicate that every 1 °C increase in average nighttime temperature during the growing season can cause up to 10% reduction in yield (Cheng et al. 2010; Mohammed and Tarpley 2010; Impa et al. 2021). Rice is particularly sensitive to HNT stress during grain development, mainly due to reduced fertility and grain weight under HNT (Morita et al. 2005; Makino et al. 2020; Dhatt et al. 2021).

Grain yield is a quantitative trait determined by number of panicles, grains per panicle, and grain weight. Grain weight has a plastic relationship with grain number per plant as increasing grain number usually decreases single grain weight (SGW) due to resource limitations (Acreche and Slafer 2006; Li et al. 2019a; Molero et al. 2019; Rivera-Amado et al. 2019; Calderini et al. 2021). In rice, forward and reverse genetic approaches have identified several genes that control grain weight under optimal conditions (Azizi et al. 2019; Li et al. 2019b; Chen et al. 2021). Genome-wide association studies (GWAS) have identified natural alleles associated with grain weight under optimal conditions (Huang and Han 2014). For instance, rice *SQUAMOSA promoter-binding protein-like 16* (*OsSPL16*)/*Grain weight 8* (*GW8*) enhances grain size and yield by promoting cellular proliferation and grain filling (Wang et al. 2012, 2015). *OsSPL14/IDEAL PLANT ARCHITECTURE1* (*IPA1*) has been proposed as a “revolutionary gene” for its role in rice yield improvement as higher expression of *IPA1* results in fewer unproductive tillers, bigger panicles along with higher grain number and weight (Wang and Wang 2017; Duan et al. 2019; Song et al. 2022). Other signaling pathways known to regulate grain weight and size include ubiquitin-proteasomal, G-protein signaling, mitogen-activated signaling, and phytohormone-mediated signaling (Chen et al. 2021). Among phytohormones, several genes involved in auxin, brassinosteroids, and cytokinin signaling have been identified as regulators of grain weight and size. *Thousand-grain weight 6* (*TGW6*), an IAA (indole-3-acetic acid)-glucose hydrolase, enhances the source-to-sink translocation by increasing free-IAA in developing endosperm (Ishimaru et al. 2013). *OsTAR1*, *OsYUC9*, and *OsYUC11* also positively regulate IAA accumulation in endosperm, which is required for normal starch accumulation (Abu-Zaitoon et al. 2012; Xu et al. 2021b). Cytokinins regulate both grain number and grain weight in rice (Ashikari et al. 2005; Xiao et al. 2019; Wang et al. 2020). Cytokinins accumulate at high level in grains shortly after fertilization during coenocytic phase of endosperm development (Bennett 1973; Zhang et al. 2008). Reduced expression of *cytokinin oxidase* (*OsCKX2*), a cytokinin degradation enzyme, increases panicle branching and hence, grain number in rice (Ashikari et al. 2005; Jameson and Song 2016; Chen et al. 2020). *Dense and erect panicle 1* (*DEP1*), a heterotrimeric G protein, also positively regulates grain weight by promoting auxin and cytokinin accumulation in developing grains (Zhang et al. 2019). Lonely Guy (LOG) family genes are proposed to encode cytokinin-activation enzymes (Kuroha et al. 2009). These genes have been reported to regulate inflorescence development in both rice and Arabidopsis (Kurakawa et al. 2007; Kuroha et al. 2009; Jameson and Song 2016). Several reports indicate that cytokinin directly regulates the heat stress tolerance in wheat and rice (Cao et al. 2010; Wang et al. 2012; Černý et al. 2014; Li et al. 2021a). Although these studies have improved molecular understanding of grain weight regulation, the role of these genetic determinants in grain weight regulation under HNT remains less explored.

Compared to HNT, our molecular and physiological understanding of high day and night temperature is more advanced (Jagadish et al. 2015, 2021; Li et al. 2015; Chiluwal et al. 2020; Poidevin et al. 2021; Schaarschmidt et al. 2021). For instance, some rice accessions escape the negative effects of short-term heat stress on pollen viability by initiating flowering during cooler hours of the day (Hirabayashi et al. 2015). Heat stress during early development stages can reduce grain weight by altering the timing of endosperm cellularization (Chen et al. 2016; Paul et al. 2020b). Disruption of starch metabolism and endoplasmic reticulum pathways deteriorate the grain quality under heat stress (Yamakawa et al. 2007; Han et al. 2012; Lo et al. 2016; Ishimaru et al. 2019; Sandhu et al. 2021). Further, heat-tolerant rice cultivars accumulate heat shock protein at a more rapid rate than heat-sensitive cultivars during grain development (Liao et al. 2014; Lin et al. 2014; Liu et al. 2021). Recent studies have identified HNT-susceptible and HNT-resilient accessions (Bahuguna et al. 2017; Kadam et al. 2018; Dhatt et al. 2021). It has been proposed that cultivars with increased nighttime respiration rates under HNT are more prone to yield losses. The smaller diurnal temperature range under HNT can also impact source-to-sink translocation (Xu et al. 2021a). For instance, an HNT-susceptible accession, “*Gharib*”, has reduced grain weight and filling, lower translocation of nitrogen and nonstructural carbohydrate from vegetative tissues to developing grains under HNT conditions (Bahuguna et al. 2017; Xu et al. 2021a).

Recent characterization of natural variation in HNT response has identified a grain-specific gene, *Fertilization Independent Endosperm 1* that regulates grain width under HNT (Dhatt et al. 2021). Several other quantitative trait loci (QTL) associated with grain yield components in rice under HNT have also been identified. These QTL studies imposed a longer-term HNT stress initiated during panicle initiation stage thus entailing cumulative variation for both pre- and post-zygotic stage mechanisms for HNT response (Bheemanahalli et al. 2021; Kumar et al. 2021). In this study, we specifically aimed to identify the genetic determinants of SGW under a post-zygotic HNT stress. We have identified several loci that are associated with SGW and focused on a region (*SGW1*) that is detected for SGW only under HNT stress. We functionally validated a putative cytokinin-activating gene, *LONELY GUY-Like 1* (*LOGL1*), as a candidate for regulating natural variation in grain weight under both control and HNT stress. Our phenotypic characterization of *logl1*-mutant and *LOGL1*-OE lines support the role of this gene as the basis of HNT variation underlying the *SGW1* locus.

## Results

### SGW trait under HNT stress

**Figure 1.**
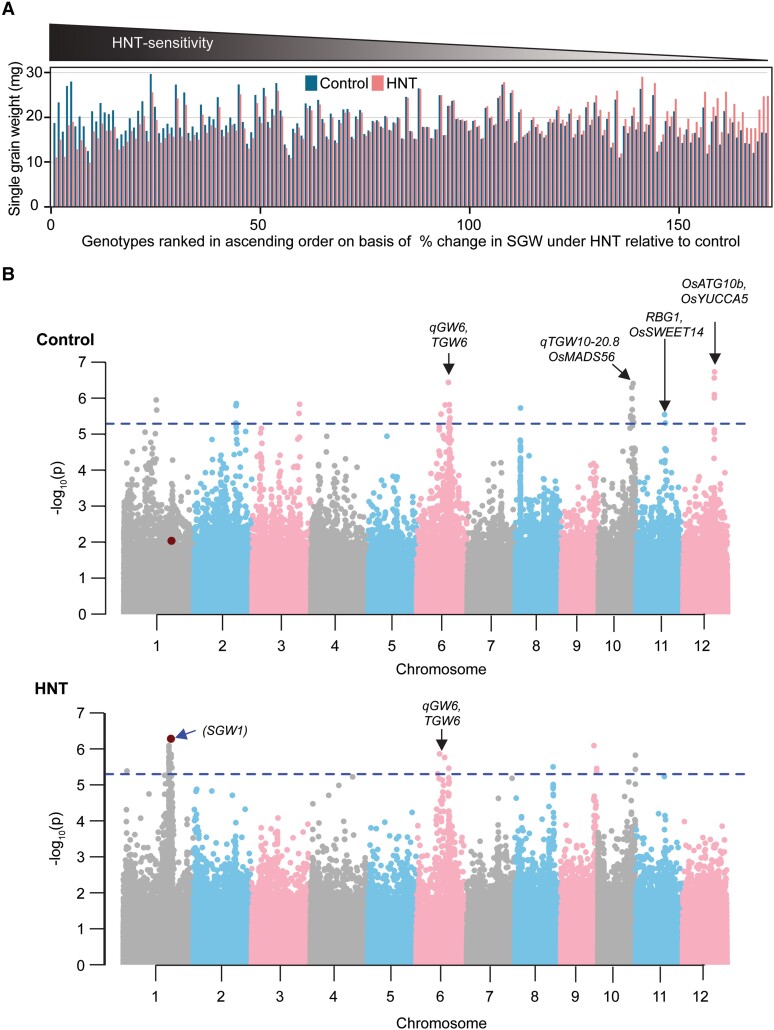
GWA analysis for SGW under control and HNT conditions in rice. **A)** Natural variation in SGW (bars) under control (blue) and HNT (red) conditions among RDP1 accessions. Accessions are arranged in ascending order (left to right) based on the percentage change in SGW of HNT-treated grains relative to corresponding controls. **B)** Manhattan plots from GWA analysis for SGW under control and HNT. Significant (*P* < 3.3 × 10^−6^ or −log10(*P*) > 5.4) SNPs are above threshold (dotted blue line). The blue arrow indicates the most significant SNP (*SGW1*) for SGW under HNT. *SGW1* is represented by a maroon dot in control and HNT Manhattan plots. Previously known genes/QTLs that co-localized with control and HNT GWA peaks are mentioned.

To determine the impact of HNT on grain weight, we imposed an HNT stress (30/28 °C) during grain development on a diverse set of rice accessions (Huang et al. 2010; Ali et al. 2011; Eizenga et al. 2014) (Supplementary Table S1). We used SGW from primary panicles as the main yield component for HNT resilience in this study. We observed considerable variation in SGW across the accessions under both control (range = 10 to 29.7 mg) and HNT (range = 8.31 to 29.1 mg) treatments (Fig. 1A). Next, we examined the percentage change in SGW of HNT-treated grains relative to control grains for the 179 accessions that were common between control and HNT. We filtered 62 accessions with more than 5% decrease (referred to as sensitive) and 49 accessions with more than 5% increase (resilient) in SGW under HNT stress compared to corresponding grains from control plants (Supplementary Table S2). Given the plastic relationship between grain number and weight in rice, we asked if higher SGW for the resilient accessions under HNT stress was due to reduced fertility (Supplementary Fig. S1A), hence lower grain number resulting in redirection of additional resources to remnant grains. We selected sensitive and resilient accessions as defined by the 5% SGW threshold and examined the panicle-level fertility of these accessions. We did not find any association (Fisher's exact test *P*-value = 0.256, Supplementary Table S2, and Supplementary Fig. S1A) between the primary panicle fertility and SGW response to HNT stress. In fact, 29 of the 49 HNT-resilient accessions maintained their fertility level under HNT stress (Supplementary Table S2). Next, we categorized these accessions based on SGW and fertility under HNT (Supplementary Fig. S1B) and identified 16 superior accessions with maximum SGW (>20 mg) and fertility (>90%) under HNT (Supplementary Table S3). Similarly, 21 inferior accessions had low SGW (<15 mg) and fertility (<70%) under HNT (Supplementary Table S3). Inferior accessions were predominantly from *Indica* and *Aus*, and superior accession list was populated by temperate japonicas (Supplementary Fig. S1C). This suggested that the phenotypic variation for the SGW trait in rice is strongly regulated at subpopulation level.

### Genome-wide association analysis for SGW under HNT

We next performed genome-wide association (GWA) analysis on the SGW trait under control and HNT stress separately. This yielded a total of 64 significant single nucleotide polymorphic sites (SNPs) associated with SGW, from which 32 were HNT-specific, 30 were control-specific, and 2 SNPs were detected under both control and HNT (Fig. 1B, Supplementary Fig. S2, and Supplementary Table S4). These significant SNPs localized to 8 and 6 distinct regions for HNT and control treatments, respectively (Supplementary Table S4). A significant peak/locus on chromosome 6 (referred as *qSGW6*) detected under both control (15 to 20.5 Mb) and HNT (13 to 20 Mb), co-localized with a previously identified QTL for grain weight, *qGW6* (Ngu et al. 2014) (Supplementary Table S4 and Fig. 1B). Within this peak (referred as *qSGW6*), SNP-6.17579336 (*sSGW6.1*), and SNP-6.20150777 (*sSGW6.2*) showed significant association with SGW under both control and HNT conditions (Supplementary Table S4). Notably, *sSGW6.2* is ∼5 Mb upstream of *TGW6*, which regulates rice grain weight by controlling IAA supply in developing endosperm (Ishimaru et al. 2013). The most significant SNP (SNP-12.19515276, −log_10_(*P*) = 6.74) under control conditions was within a peak (19.3 to 19.5 Mb) detected on chromosome 12. We scanned this region for the presence of previously characterized genes and found that a gene involved in IAA synthesis, *OsYUCCA5* is localized within 250 kb downstream of this lead SNP (Li et al. 2021b). Most of the other genes in this region were annotated as either expressed protein or transposable elements. Another control-specific set of SNPs was detected on Chromosome 11. The 2 top SNPs are located upstream (SNP-11.17221237, position = 17707382) and downstream (SNP-11.17705601, position = 18191744) of *Rice Big Grain 1* (*RBG1*). *RBG1* promotes cell division resulting in increased organ size (Lo et al. 2020). Additionally, *OsSWEET14* is located 20 kb downstream of SNP-11.17705601. *OsSWEET14* encodes for a sucrose efflux protein that regulates supply to developing endosperm during grain filling stage (Fei et al. 2021). We found that a large peak on chromosome 10 colocalized with recently identified QTL for thousand-grain weight, *qTGW10.20.8* (Zuo et al. 2021). The candidate gene underlying *qTGW10.20.8*, *OsMADS56* positively regulates thousand-grain weight in rice (Zuo et al. 2021). Collectively, colocalization of several significant SNPs with previously known grain size and weight regulating genes validates our experimental approach.

**Figure 2.**
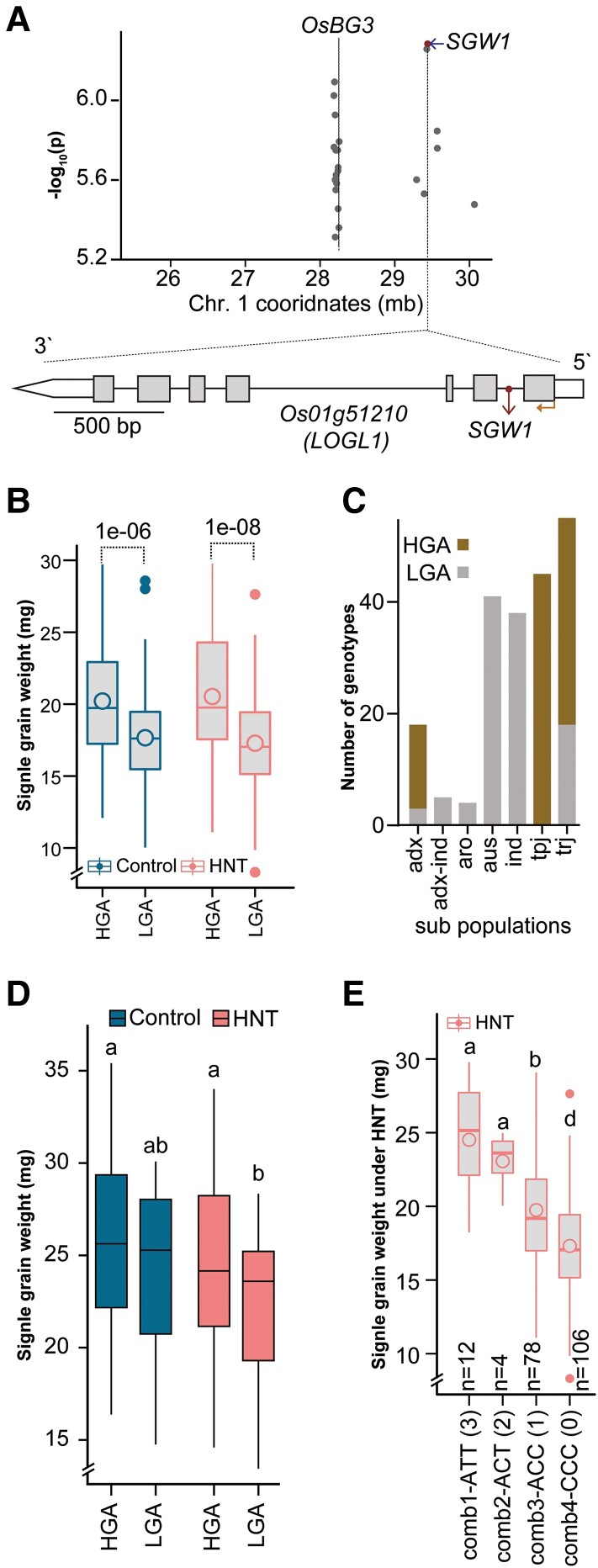
*LOGL1* could be a determinant of SGW under HNT. **A)** Zoom-in plot showing the 2 most significant peaks on Chromosome 1 under HNT. HNT-specific SNP (maroon dot) *SGW1* localized to the first intron (maroon arrow) of a candidate gene, *Os01g51210* (*LOGL1*). A genomic map (on reverse strand) of *LOGL1* containing exons (gray rectangles), untranslated regions (white rectangles), introns (line), and start codon (golden arrow) is shown in lower panel. **B)** Boxplot showing allelic effect of *SGW1* locus on SGW. Here, HGA and LGA represent 2 allelic groups at *SGW1*. *P*-values (indicated by text) represent significant difference (*t*-test) between SGW of HGA (*n* = 94) and LGA (*n* = 106) within a treatment (control or HNT). **C)** Subpopulation level distribution of HGA and LGA alleles in RDP1. **D)** The SGW of HGA (*n* = 58, 2 plots per genotypes) and LGA (*n* = 20, 2 plots per genotypes) accessions under ambient (control) and post-flowering HNT stress conditions in field high tunnel greenhouses. Different letters represent significant differences (LSD test, *P* = 0.05). **E)** Box plots showing impact of allelic combinations for the lead HNT-specific SNP (*SGW1*) and common (detected under both control and HNT) SNPs (*sSGW6.1 and sSGW6.2*) on Chromosome 6 on SGW under HNT. Here, ATT, ACT, ACC, and CCC represent 4 different allelic combinations (comb); *n* indicates number of accessions in each comb. The text in parentheses indicates number of favorable alleles in each combination. Different letters represent significant differences (*t*-test, *P* = 0.05). In box plots **B)**, **D)**, **E)**, center line, median; box limits, upper and lower quartiles; whiskers, 1.5× interquartile range; filled circles, outliers and unfilled circle inside box, mean.

The most prominent peak detected for SGW under HNT was on Chromosome 1 in a region composed of multiple haploblocks (Fig. 1B and Supplementary Fig. S3). This peak resolved into 2 distinct regions (Fig. 2A). A 71.25 kb region in this peak has 16 significant SNPs (Fig. 2A). We found that *Big Grain 3* (*BG3*) is located ∼190 kb upstream of this block. *BG3* positively regulates grain size and weight by modulating the long-distance cytokinin transport (Xiao et al. 2019). We found another distinct region (*qSGW1*), which was populated by 6 SNPs (Fig. 2A). The lead HNT-specific SNP was SNP-1.29438503 (hereafter, *SGW1*; position = 29439549, −log_10_(*P*) = 6.284) which explained 10.5% (*R*^2^ = 0.105) of phenotypic variation under HNT stress only (Fig. 2A and Supplementary Fig. S3). We next calculated the linkage disequilibrium (LD) between the SNPs near *SGW1*, including the lead SNP for *qSGW1* (Supplementary Fig. S3 and Supplementary Table S5). The LD analysis indicates that the lead SNP for *qSGW1* has large LD with the 2 nearby SNPs (SNP-1.29427477 and SNP-1.29392387) and extent of LD decreases as they become more distant. Based on the lead, HNT-specific SNP, we labeled the 2 allelic groups for *SGW1* as heavy-grain accessions (HGA), which have the “A” allele, and light-grain accessions (LGA), corresponding to “C” allele. The mean SGW of HGA was 18% higher than LGA under HNT stress (Fig. 2B). Under control temperatures, this allelic difference was 14%. We found that all *indica* (*n*^LGA^ = 38) and *aus* (*n*^LGA^ = 41) accessions have the LGA allele and all *temperate japonica* accessions (*n*^HGA^ = 45) have the HGA allele (Fig. 2C). However, *Tropical japonica* accessions have both LGA (*n*^LGA^ = 18) and HGA (*n*^HGA^ = 37) alleles. Overall genetic background differences between *indica* and *temperate japonica* can confound assessment of the contribution of HGA and LGA alleles toward SGW (Lu et al. 2013). Therefore, we also examined the allelic effect of HGA and LGA among different subpopulations (Supplementary Fig. S4A). In *tropical japonicas* that carry both alleles, SGW of HGA is significantly higher than LGA, but only under HNT. The reason for detection of *SGW1* only under HNT even though the allelic groups differ in control conditions as well could be a consequence of correcting for population structure during the association analysis. We next tested the allelic effect of the *SGW1* in grain weight regulation under stress by performing HNT treatment in field conditions using heat tents (Quiñones et al. 2023). We imposed an HNT stress on a subset of Rice (*O. sativa*) Diversity Panel 1 (RDP1) accessions by heating the tents in the field to 4 °C above the nighttime ambient (control) temperature during nighttime (Supplementary Fig. S4B). Because of the wide range in initiation of flowering among these accessions, we specifically targeted 39 *tropical japonica* accessions that initiated flowering in an overlapping window during grain development when HNT stress was imposed for this analysis. A corresponding set of accessions were grown in control tents with no heating during nighttime. We found that the HGA allelic group has a higher SGW under HNT relative to the LGA group (Fig. 2D). Under control conditions, SGW of HGA was not significantly different than LGA These results indicate that *SGW1* regulates grain weight variation under HNT stress in both greenhouse and field experimental set-up with a more significant allelic difference observed under HNT stress conditions.

### Stacking favorable alleles improves SGW trait under HNT stress

Next, we determined the SGW outcome of stacking favorable alleles of the lead HNT-specific SNP (*SGW1*) and common (detected under both control and HNT) SNPs on Chromosome 6 (*qSGW6*). These 3 markers were *SGW1*, *sSGW6.1*, and *sSGW6.2*, and the corresponding favorable (conferring higher SGW) alleles were “A”, “T”, and “T”, respectively (Supplementary Fig. S5A and Supplementary Tables S1 and S4). We obtained 4 allelic combinations (comb1, comb2, comb3, and comb4) and analyzed their SGW under control and HNT (Fig. 2E and Supplementary Fig. S5B). This analysis indicated that grain weight under both conditions is significantly low for comb3 and comb4 with the presence of the unfavorable allele for *SGW1*and *sSGW6.2*, respectively. Frequency of comb1 with the 3 favorable alleles is very low in RDP1 but has the highest SGW under both control and HNT conditions. This suggests that developing germplasm with higher frequency of comb1 allelic combination can improve SGW resilience to HNT stress.

### Natural variation in *LOGL1* regulates SGW under HNT

**Figure 3.**
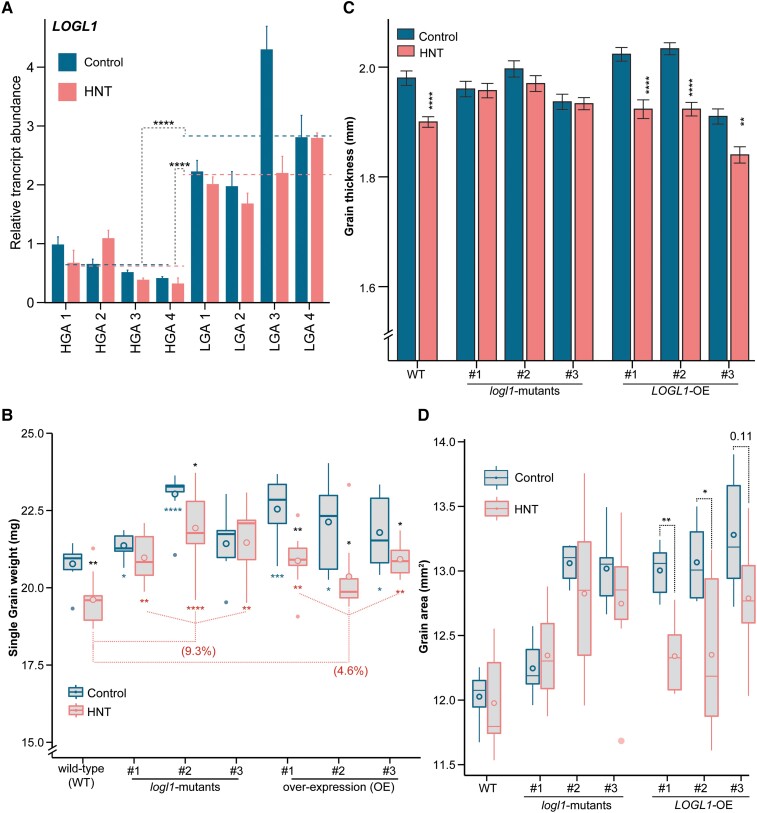
*LOGL1* alters the HNT sensitivity of SGW. **A)** Allelic variation in *LOGL1* transcript abundance (mean+/−SE, *n* = 2 biological replicates with more than 25 seeds per replicate obtained from 3 to 4 plants) among 4 HGA and LGA group accessions under control and HNT. The dotted lines represent average of 4 accessions within an allelic group under control (blue) and HNT (red). The *t*-test (****, *P*-value < 0.0001) was used to compare average transcript abundance of *LOGL1* in HGA to LGA within control and HNT. The transcript abundace in grains at 2 days after fertilization was quantified using reverse transcription quantitative PCR (2^−ΔΔCT^ method). The 4 accessions from HGA are Kitaake, NSFTV-113, NSFTV-56, and NSFTV-303, and from LGA are NSFTV-231, NSFTV-251, NSFTV-258, and NSFTV-24. **B)** to **D)** SGW **B)**, grain thickness (mean ± SE) **C)**, and grain area **D)** of WT, *logl1-*mutants and OE lines under control or HNT treatments. In box plots **B)**, **D)**, center line, median; box limits, upper and lower quartiles; whiskers, 1.5× interquartile range; filled circles, outliers and unfilled circle inside box, mean. In **B)**, **C)**, and **D)**, Significant difference (*t*-test, *n* = 9) between C and HNT within each genotype is indicated by black asterisks. In **B)**, *t*-test was used to compare individual *logl1* and OE events to WT within control (blue asterisks) or HNT (red asterisks). In **B)**, (%) percentage difference between *logl1* (averaged for 3 events) and OE (averaged for 3 events) compared to WT within C (blue) or HNT (red) is indicated by dotted lines. In **A)** to **D)**, (*, *P*-value < 0.1; **, *P*-value < 0.01; ***, *P*-value < 0.001; and ****, *P*-value < 0.0001).

We next examined the genes in proximity to the lead SNP, *SGW1*. This SNP localized to the first intron of *LOGL1* (*Os01g51210*) (Fig. 2A). We examined 30 genes (including 6 transposable elements) within a 100 kb interval of this SNP (Supplementary Table S6). Since the HNT treatment is imposed during grain development, we excluded 16 genes for which we could not detect transcript-level signal in developing grains from multiple transcriptome datasets. We found *LOGL1* to be highly active, with increased transcript abundance during reproductive development (GSE6893; Supplementary Fig. S6A) (Jain et al. 2007). We evaluated the transcript level allelic variations for the remaining 14 genes (with transcripts detected in grains) in seedling transcriptome data for 98 (46 LGA, 52 HGA) RDP1 accessions (GSE98455; Campbell et al. 2020). We reasoned that any allelic variation in transcript abundance in developing grains is also likely to be detected in other tissues and developmental stages. *LOGL1* exhibited the highest allelic variation among the 14 genes, with ∼2-fold higher transcript abundance, in LGA relative to HGA (Supplementary Fig. S6B). We further validated allelic variation in *LOGL1* transcript accumulation in developing grains at 2 days after fertilization (DAF) (Fig. 3A). The HGA group showed lower transcript abundance of *LOGL1* than LGA group. We also analyzed the expression of other nearby genes including, *OsbHLH118*, *OsTRBF3*, and *CK1* in HGA and LGA group accessions under control and HNT stress in grains (Supplementary Figs. S7). Expression of *OsbHLH118* is very low for reliable detection in grains at 2 DAF, which is consistent with public microarray dataset shown in Supplementary Table S6. Transcript abundance of *OsTRBF3* and *CK1* kinase in HGA and LGA allelic groups do not show a consistent difference. Collectively, the localization of the lead SNP in the intron of *LOGL1* and a significant allelic expression level difference suggests that *LOGL1* is the candidate gene underlying *SGW1.*

The members of the LOG gene family encode for cytokinin-activating enzymes that convert inactive cytokinin nucleotides to active free-base forms, *N6*-(Δ2-isopentenyl) adenine (iP), and *trans*-zeatin (tZ) (Kurakawa et al. 2007; Kuroha et al. 2009). An LOG-family gene, *LOG* regulates shoot apical meristem and inflorescence development in rice (Kurakawa et al. 2007). Therefore, we decided to further characterize *LOGL1* as the candidate gene underlying *SGW1*. For this, we generated constitutive overexpression (OE) and CRISPR-Cas9 (CR)-based (referred as *logl1*) mutants in rice cv Kitaake, which carries the HGA allele (“A” allele) at the *SGW1* locus (Jain et al. 2019) (Supplementary Fig. S8, A and B). Three homozygous *logl1-*mutants, each carrying a different mutation, had premature stop codons and ∼2.5-fold (log_2_) reduction in transcript accumulation (Supplementary Fig. S8, B and C). OE lines had 2.5- to 5-fold (log_2_) higher transcript abundance in 2 DAF grains, a development stage when *LOGL1* is highly expressed and coincides with HNT stress imposition in our diversity panel screening (Supplementary Fig. S8C).

Next, we investigated the phenotypic response of *logl1*, OE and wild type (WT) lines under control and HNT conditions, where HNT treatment was imposed throughout the grain development (Supplementary Fig. S9A). Compared to WT, all *logl1-*mutants showed higher SGW under HNT and 2 (#1 and #2) of the 3 *logl1-*mutants exhibited higher SGW under control (Supplementary Table S7 and Fig. 3B). Surprisingly, 2 OE lines (#1 and #3) under HNT and all OE lines under control also maintained significantly higher SGW than corresponding WT grains (Supplementary Table S7 and Fig. 3B).

However, compared to corresponding control-treated grains, each OE mutant showed a significant reduction in SGW ranging from 4% to 10.8% in response to HNT. Grains from WT exhibited a 5% decline under HNT (Fig. 3B). Under HNT, OE plants also showed significant reduction in grain size parameters including, length, width, area, and thickness compared to corresponding control grains (Fig. 3, C and D and Supplementary S9B). We found that WT grain thickness significantly reduced under HNT relative to control. In contrast, SGW and grain size of 2 *logl1* lines under HNT is similar to the corresponding control-treated plants, except for reduction in SGW of *logl1*#2 (Fig. 3, B to D and Supplementary S9B). For all *logl-*mutants, average SGW is ∼5% higher under control conditions and ∼9% higher under HNT stress when compared to corresponding WT (Fig. 3B and Supplementary Table S7). We also evaluated grain chalkiness and found that HNT had similar impact on grain chalkiness in all genotypes (Supplementary Fig. S9C). Taken together, these results suggest that increased abundance of *LOGL1* in developing grains of OE lines increases HNT sensitivity of SGW, while all *logl1-*mutants had significantly higher SGW than WT under HNT stress.

### *LOGL1* regulates tillering and yield-associated traits

**Figure 4.**
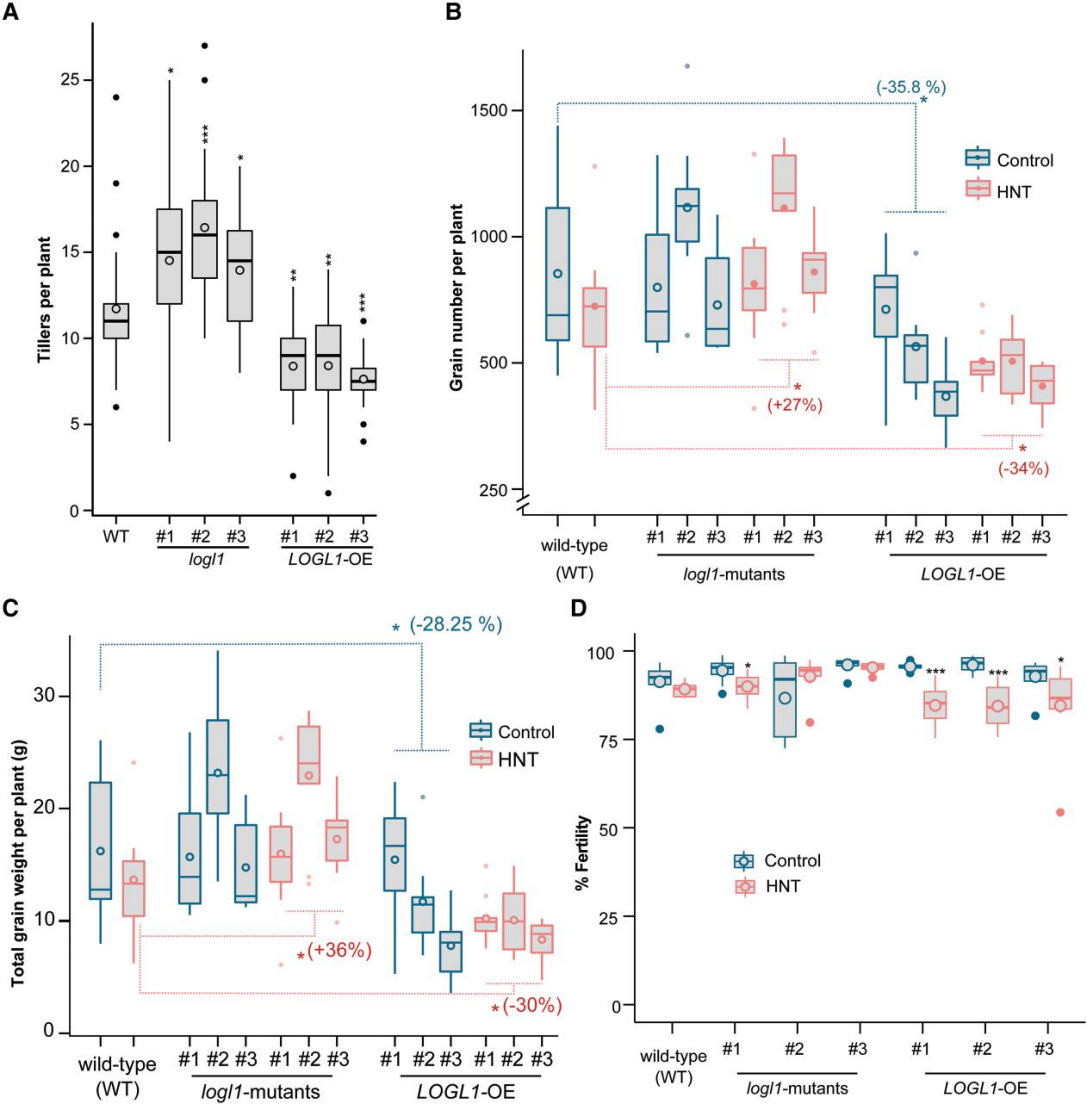
*LOGL1* misregulation alters yield parameters. **A)** Number of tillers for *logl1-*mutants, OE, and WT plants (*n* = 24) at flowering phase under control conditions. The asterisks represent significant difference (*t*-test) compared to WT. **B)** Grain number and **C)** total grain weight per plant, and **D)** fertility of WT, *logl1-*mutants and OE lines under control (C) or HNT treatment. In **A)** to **D)** (*, *P*-value < 0.1; **, *P*-value < 0.01; ***, *P*-value < 0.001; and ****, *P*-value < 0.0001). In **B)** and **C)**, *logl1* (averaged for 3 events) and OE (averaged for 3 events) were compared to WT within C (dotted blue line) or HNT (dotted red line) using *t*-test. In **D)**, significant difference (*t*-test) between control and HNT within each genotype is indicated by black asterisks. In **B)** to **D)**, *n* = 9. In box plots **A)** to **D)**, center line, median; box limits, upper and lower quartiles; whiskers, 1.5× interquartile range; filled circles, outliers and unfilled circle inside box, mean.

We observed that both OE and *logl1*-mutants had higher SGW than WT. Grain weight is a complex trait regulated by multiple genes and has a plastic relationship with grain number. To evaluate if *LOGL1* regulates grain number and other yield parameters, we determined the tiller number, grain number, grain weight per plant and fertility for WT, *logl1*-mutants and OE lines. We found that *logl1* lines had significantly higher tiller number, while OE lines had reduced tiller number compared to WT under control conditions (Fig. 4A). These results corresponded to lower grain number and grain weight per plant of OE compared to WT under control conditions (Fig. 4, B and C and Supplementary Table S7). We found that 2 OE lines (OE#2 and OE#3) showed significantly lower grain number and showed reduction in total grain weight per plant than WT under control conditions. However, under HNT, all the OE lines had significantly lower grain number and grain weight per plant compared to HNT-treated WT (Fig. 4, B and C and Supplementary Table S7). On the contrary, the grain number and total grain weight per plant for individual *logl1*-mutants (except, *logl1*#2) was not significantly different than WT plants under control or HNT stress (Fig. 4, B and C and Supplementary Table S7). Interestingly, the higher HNT sensitivity of OE lines is also reflected as reduced fertility under HNT (Fig. 4D). We measured the whole plant fertility of control and HNT-treated plants and found that OE lines showed significant reduction in fertility under HNT compared to corresponding controls. These results suggest that the reduced grain number in OE lines is limiting the sink capacity and hence redirecting the available carbohydrates to a smaller number of grains which exhibit higher SGW in OE lines relative to WT. This redirection of carbon resources to a reduced sink capacity resulting from a smaller number of grains is a compensatory response between the 2 yield components (Figs. 3B and 4B). Overall, the phenotypic analysis indicates that *LOGL1* regulates important agronomic traits in rice.

### *LOGL1* regulates reactive oxygen species pathway under HNT

**Figure 5.**
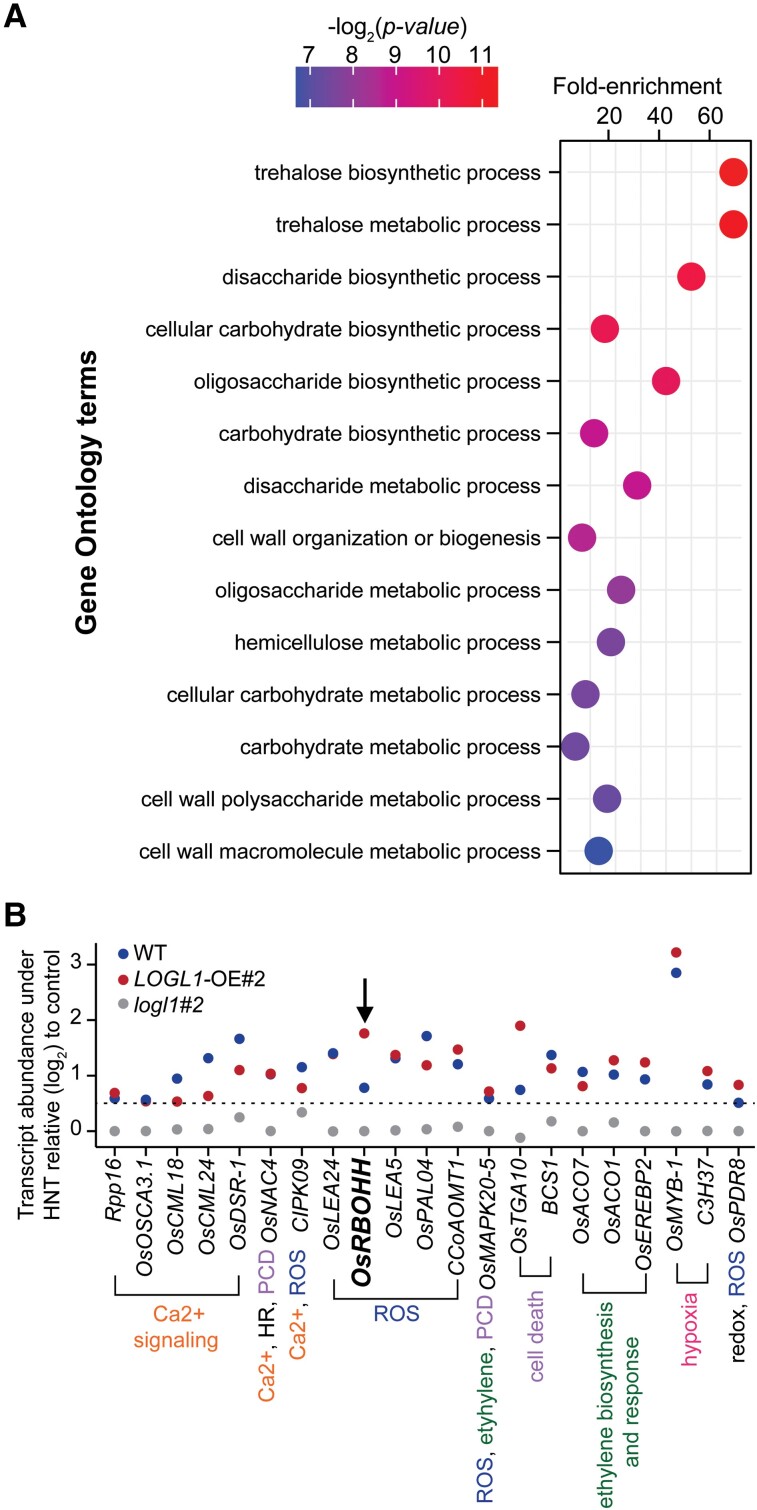
Transcriptome analysis of grains under control and HNT. **A)** GO-based biological processes enriched among genes (*n* = 33) which were upregulated by HNT (compared to respective control) for logl1#2 but did not change for OE and WT. **B)** Genes involved in reactive oxygen species (ROS) production, ethylene biosynthesis, HR, PCD, and Ca2^+^-signaling that are significantly upregulated [adjusted-*P* < 0.1, log_2_(foldchange) > 0.5] by HNT (relative to respective controls) in OE#2 and WT, but did not change in *logl1#*2 [adjusted-*P* > 0.1, log_2_(foldchange) < 0.5].

We next sought to determine the impact of *LOGL1* misregulation on the transcriptome of 2 DAF grains in *logl1* (#2), OE (#2), and WT. We identified differentially expressed genes (DEGs) between HNT stress and control for *logl1*, OE, and WT and found that *logl1* has a lower number of HNT-regulated DEGs than OE and WT (Supplementary Fig. S10A). A less perturbed transcriptome of *logl1* under HNT stress is consistent with lower HNT sensitivity of *logl1* grains. We identified 33 genes that are upregulated by HNT in *logl1* but did not differentially respond to HNT in OE and WT grains (Supplementary Fig. S10B). These genes showed strong enrichment of trehalose metabolism and carbohydrate biosynthesis (Fig. 5A). Notable among these were rice *trehalose-6-phosphate phosphatase 1* (*OsTPP1*), *OsTPP7*, *basic helix-loop-helix protein 006* (*bHLH006*), *a cytochrome P450 enzyme* (*CYP94C2b*), and *JASMONATE ZIM DOMAIN 9* (*OsJAZ9*) (Supplementary Fig. S10B). Among these, *OsTPP1*, *OsTPP7*, and *OsJAZ9* are known to positively regulate stress tolerance in rice at seedling stage (Pramanik and Imai 2005; Ge et al. 2008; Zhang et al. 2017).

We also identified 72 genes that are upregulated by HNT stress for WT and OE but did not change significantly in *logl1* grains (Supplementary Fig. S10C and Supplementary Tables S8 to S9). These included genes that are involved in ROS (reactive oxygen species) production, ethylene biosynthesis, hypersensitive response (HR), program cell death (PCD), and Ca^2+^-signaling (Fig. 5B and Supplementary Tables S8 to S9). Ca^2+^-induced NADPH oxidases and ROS-activated Ca^2+^ channels form self-amplifying “ROS-Ca^2+^ hub”, which is central to HR-induced PCD (Carimi et al. 2005; Demidchik et al. 2017). Furthermore, ethylene overproduction enhances hypoxic responses and mediates autophagy likely via ROS signaling (Hartman et al. 2021). We found that HNT-induced accumulation of *respiratory burst oxidase homolog* (*OsRBOHH*), a NADPH oxidase (Yamauchi et al. 2017), was highest for OE followed by WT. However, *OsRBOHH* transcript abundance did not change under HNT for the *logl1*-mutant.

### *LOGL1* alter circadian clock genes and downstream thiamin synthesis

**Figure 6.**
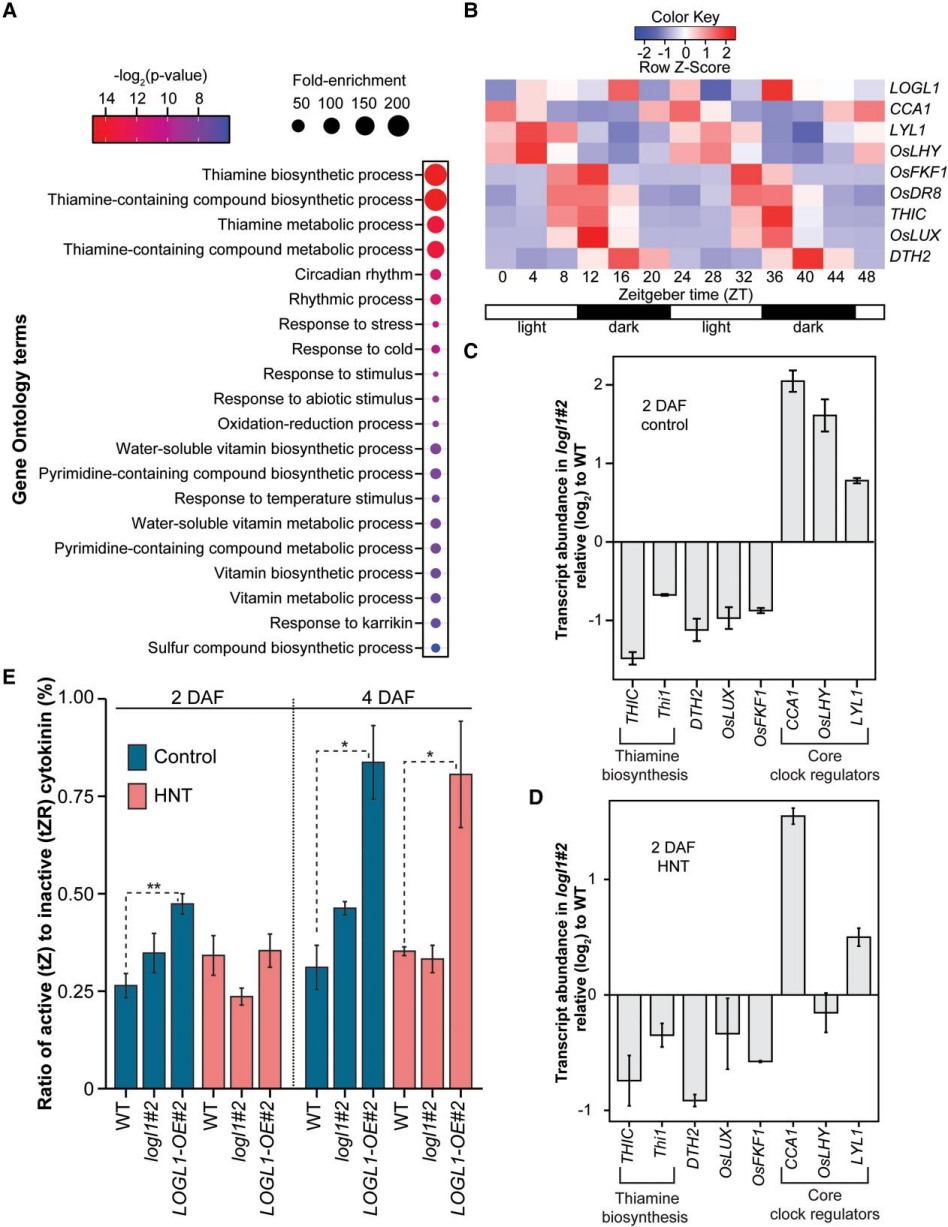
*LOGL1*-misregulation alter the clock and thiamin biosynthesis genes. **A)** GO-based on top 20 biological processes enriched among genes (*n* = 80) with downregulation in *logl1#*2 under control conditions. **B)** Rhythmic expression of thiamin and clock genes in rice publicly available diurnal dataset. **C)** and **D)** Altered expression of thiamin biosynthesis and core circadian-related genes in *logl1#*2 relative to WT [log_2_(foldchange), mean ± SE] **C)** control and **D)** HNT. **E)** The ratio of active (tZ) to inactive (tZR) cytokinin in control and HNT-treated grains and 2 and 4 days after fertilization (DAF). Error bars represent mean ± SE, *n* = 2 biological replicates with more than 25 seeds per replicate obtained from 3 to 4 plants. Here, *P < 0.1, **P < 0.01; *t*-test was used to compare *LOGL1-*OE#2 and *logl1#*2 to WT (Mockler et al. 2007).

To identify *LOGL1-*mediated regulatory pathways, we performed Gene Ontology (GO) term analysis and found that genes involved in diurnal/circadian regulation, light stimulus, and vitamin B1 (thiamin) synthesis are significantly enriched among DEGs between *logl1#*2 and WT under control conditions (Supplementary Table S10). In particular, expression of genes involved in thiamin biosynthesis-related genes was downregulated in *logl1* compared to WT (Fig. 6A). The co-enrichment of circadian and thiamin pathways among the DEGs is notable as 2 of the clock genes, *Circadian Clock Associated1* (*CCA1*) and *Late Elongated Hypocotyl* (*LHY*) bind to *THIC* promoter and regulate its expression in a circadian manner (Bocobza et al. 2013; Noordally et al. 2020). We analyzed the expression these genes in diurnal dataset and found that expression of *CCA1* in rice peaks near start of light period, while *THIC* has highest accumulation near end of light period (Fig. 6B). Notably, transcript levels of *LOGL1* are not under circadian regulation (Fig. 6B). We found that compared to WT, *logl1* grains have lower transcript accumulation of *THIC*, *THI1*, and higher accumulation of *CCA1* and *LHY1* under both control conditions and HNT conditions (Fig. 6, C and D).

### *LOGL1-*OE alters the active cytokinin levels in developing grains

We next measured the levels of active (tZ) and inactive forms of cytokinin (*trans*-zeatin riboside, tZR) in 2 and 4 DAF developing grains of WT, a *logl1#*2 mutant and OE#2 line under control and HNT (Fig. 6E and Supplementary Fig, S11). There was no significant difference for tZ and tZR except for the increase in tZ and tZR levels in *LOGL1-*OE#2 at 2 DAF under control conditions. Given that LOG genes encode for cytokinin-activating enzymes that convert inactive cytokinin to active form, we examined the ratio of active (tZ) to inactive cytokinin in 2 and 4 DAF developing grains (Fig. 6E). *LOGL1-*OE#2 had significantly higher ratio of active to inactive cytokinin under both control and HNT conditions at 4 DAF and under control condition at 2 DAF. No difference was observed between *logl1#*2 mutant and WT. These results suggest that disruption of *LOGL1* may not be altering the levels of active cytokinin at single grain level.

## Discussion

This study examines the extent of natural variation for SGW under an HNT stress imposed to specifically coincide with grain development on the primary panicle. We found considerable variation in HNT response at SGW level. Within this variation, we found 29 accessions with significantly higher SGW under HNT stress without any increase in sterility. Stress can induce sterility leading to increased SGW as available assimilates are redirected to fewer grains (Acreche and Slafer 2006; Li et al. 2019a; Calderini et al. 2021). Previous studies have shown a negative impact of HNT stress on rice yield (Bheemanahalli et al. 2021; Kumar et al. 2021). However, the impact of any stress depends on developmental timing and duration of stress. Our experimental setup was specifically designed to capture the SGW response rather than the whole plant response for the GWA component. This approach has enabled us to track the grains that were stressed post-fertilization and uncover natural variation for HNT response at single grain level. Therefore, these results are not directly comparable to other reports, where HNT treatment was imposed and yield parameters measured for a duration that covered both pre- and post-fertilization stages of development.

Besides identifying HNT-resilient accessions, we also used GWA analysis to identify several loci that are associated with grain weight under both control and HNT. A peak on chromosome 6 was detected under both control and HNT treatments. This region was colocalized with a previously identified grain weight QTL, *GW6* (Ngu et al. 2014). The lead SNP for this locus was in the vicinity of *TGW6*, which is known to regulate thousand-grain weight in rice (Ishimaru et al. 2013). Loss of *TGW6* limits the IAA supply in developing endosperm, delaying its transition to cellularization which causes higher thousand-grain weight and grain size due higher number of endosperm cell layers (Ishimaru et al. 2013).

The most prominent HNT-specific peak localized to Chromosome 1, where the lead SNP, *SGW1*, resolved to *LOGL1.* We provide evidence that *LOGL1*, which encodes for a putative cytokinin-activating enzyme is involved in grain weight regulation under HNT stress. Although the *LOGL1-*OE line had a higher accumulation of active cytokinin in developing grains, we did not observe any differences in active cytokinin between a *logl1*-mutant and WT grains. In rice, *LOGL1* is a member of an 11 gene family that is proposed to catalyze the dephosphorylation and deribosylation of iP- and tZ-nucleotides to produce active nucleobase forms, iP and tZ (Kurakawa et al. 2007; Kuroha et al. 2009). In young grains, cytokinin levels are transiently elevated to promote nuclear and cellular divisions in the endosperm, which are directly linked to grain size and weight at maturity (Chen et al. 2016; Jameson and Song 2016; Xu et al. 2021b).

Transcript abundance of *LOGL1* was significantly higher in LGA relative to HGA. It is notable that the lead SNP (*SGW1*) falls in the first intron of the *LOGl1* gene. The first intron in plants has been associated with intron-mediated expression enhancement (Rigal et al. 2012; Back and Walther 2021). Based on these results, we postulated that higher expression of *LOGL1* in LGA-allele accessions could result in lower grain weight when compared to HGA-allele accessions. We directly tested this hypothesis by creating *LOGL1*-OE and CR-based *logl1*-mutant events. We found that OE of *LOGL1* increased the HNT sensitivity of developing grains as OE grains showed higher reduction in SGW response to HNT stress compared to mutants and WT. OE grains also showed significant reduction in grain fertility under HNT compared to control conditions. On the contrary, *logl1*-mutants had higher grain weight under HNT compared to WT. It is notable that both *logl1*-mutants and OE had higher SGW than WT under HNT. However, OE plants also produced lower number of grains and yielded lower than WT and *logl1*-mutants under both control and HNT. The lower grain number but higher SGW of OE lines is likely due to commonly observed tradeoff effect between these 2 yield components in rice (Li et al. 2023). Cytokinin levels increase in developing grains after flowering before exhibiting a decline toward grain maturation (Yang et al. 2000) and this trend in cytokinin accumulation coincides with the timeline of *LOGL1* expression in developing grains (Supplementary Fig. S6A). Therefore, it is plausible to consider that increasing cytokinin levels correspond to increased sink capacity and subsequent decline in cytokinin levels corresponds to grain filling. We found increased accumulation of active cytokinin in the OE grains, which could be contributing to the compensatory effects between grain weight and number. Further, there is evidence of increased thousand-grain weight in *LOGL5* mutants of rice (Wang et al. 2020). *LOGL5* is one of the 11 gene family members along with *LOGL1*. The higher accumulation of active cytokinin in OE grains suggest negative impact on HNT resilience. It is notable though that OE lines are under control of ubiquitous promoter. It is possible that *LOGL1* may have differential cell type specificity in regulating grain weight and HNT sensitivity.

We also observed that one of *logl1*-mutants (*logl1*#2) had significantly higher (11% increase) SGW and total grain weight per plant (43% increase) than WT under control conditions. Although *logl1*#2 mutant used for this study are from T2 generation, we have consistently observed this deviation through previous generations under control conditions. The *logl1*#2 mutant carries a single base pair insertion while the other 2 mutants result from 1 bp deletion in *logl1#*1 and a 41 bp deletion in *logl1#*3. Determining if these structural differences underlie the improved performance of *logl1*#2 would require a generation of additional events with the same or similar mutations as the *logl1*#2 mutant. Therefore, we cannot currently determine the basis of this superior agronomic phenotype of *logl1*#2.

A recent study in rice shows that HNT disrupted the expression of circadian clock genes along with photosynthetic and carbohydrate metabolism pathway-related genes (Desai et al. 2021). We found that DEGs between WT and a *logl1-*mutant were enriched in circadian and thiamin biosynthesis pathway. Thiamin is essential for carbon metabolic reactions including, glycolysis, citric acid cycle, and the oxidative pentose phosphate pathway (Boubakri et al. 2013; Bunik et al. 2013). *THIC* and *THI1* are involved in thiamin biosynthesis (Raschke et al. 2007; Bocobza et al. 2013; Garcia et al. 2014) and are under circadian regulation via *CCA1* and *LHY* (Bocobza et al. 2013; Noordally et al. 2020). Expression of *THIC* is negatively regulated by *CCA1*, which was reflected in their opposite expression trend in diurnal dataset. Our transcriptome data show that *THIC* and *THI1* have lower, while *CCA1* and *LHY1* have higher accumulation in *logl1*-mutant than WT under control conditions. Lower abundance of *THIC* and higher abundance of *CCA1* in *logl1* than WT was also observed under HNT stress. Since the circadian clock receives input from both light and temperature (Bocobza et al. 2013; Desai et al. 2021), the combination of warmer nights and suppressed *LOGL1* may cause a temporal shift in thiamin activity. Warmer nights can impact the next day's photosynthesis by delaying the activation of morning genes and photosynthetic genes (Desai et al. 2021). Further, cytokinin signaling is known to regulate circadian genes. For instance, cytokinin treatment induced the expression of morning genes, *CCA1* and *LHY* while suppressing the evening complex component, *TOC1* (Hanano et al. 2006; Zheng et al. 2006). Our transcriptome analysis suggests that disruption of *LOGL1* could be altering the HNT-induced temporal shifts in diurnal processes in a manner that may mitigate the impact of HNT on grain weight. The role of *LOGL1* in diurnal fluctuation of carbon flux in thiamin-mediated pathways will be examined in future experiments.

Under oxidative stress, levels of thiamin increase in the plants, which activates the ROS-scavenging enzymes (Tunc-Ozdemir et al. 2009). Genes involved in ROS signaling including, HR-induced PCD, Ca^2+^-induced NADPH oxidases, and ROS-activated Ca^2+^ channels were strongly upregulated by HNT in WT and OE but remained unchanged in *logl1*-mutant. Since thiamin biosynthesis genes are suppressed in a *logl1*-mutant but not in WT and the OE line, it is possible that lack of ROS-signaling-associated transcriptome response in the *logl1*-mutant could be indirectly lowering thiamin levels in *logl1* grains. *LOGL1* encodes for a cytokinin-activating enzyme and hence transcriptional response could be an indirect effect of metabolic changes in OE and mutant. Collectively, the transcriptome results suggest that *LOGL1* is playing a role in regulating diurnal carbon flux in rice and its suppression in mutants enhances carbon flux to grains as well as suppresses triggering of increased Ca^2+^ and ROS-signaling response under HNT stress. ROS genes are also diurnally regulated by *CCA1* and *TOC1* (Lai et al. 2012).

In summary, we show that *LOGL1* contributes toward genetic variation for grain weight under control and HNT conditions in both controlled and field environments. We found that seeds deficient in *LOGL1* do not trigger ROS and cell death-associated transcriptional responses under HNT stress. The genetic architecture of grain weight under HNT conditions elucidated by this work could provide improved mechanistic understanding and potential target genes for rice breeders to enhance climate resilience.

## Materials and methods

### Plant material, HNT treatment, and GWAS

RDP1 accessions were screened for natural variation in SGW under control and HNT stress. Six uniformly germinated seedlings per accession were transplanted into pots (4 inches) containing natural soil and pots for each genotype were randomly arranged across the greenhouse to avoid spatial effect. Until flowering, plants were grown in a controlled greenhouse with 28/23 ± 1.5 °C temperature, 16/8 h light/dark and 55% to 60% relative humidity. During flowering, when the primary panicle reached ∼50% flowering, half of the plants for each accession were moved to HNT (30/28 ± 1.5 °C, light/dark 16/8 h) greenhouse and remaining half were maintained in control conditions. During post-flowering HNT treatment phase, the plants in HNT and control greenhouses were spread out uniformly to avoid any border effects. The HNT treatment was maintained until maturity. Mature dehulled grains from primary panicles were used for SGW analysis. SGW data were further analyzed in R software (R Core Team 2019) to obtain adjusted means for each accession across replications using the following statistical model (Dhatt et al. 2021).


$$y_{ik}=\;\mu +g_{i}+r_{k}+\varepsilon_{ik}\;$$

Here, $y_{ik}$ is SGW for *k*th replication in *i*th accession, *μ* is the intercept, $g_{i}$ is the effect of *i*th accession, $r_{k}$ is the effect of *k*th replication, and $\varepsilon_{ik}$ is the residual error associated with each SGW observation. GWA analysis was carried out as described previously (Dhatt et al. 2021). Briefly, 700k SNP markers were filtered for missing data (<20%) and minor allele frequency (<5%), and 411,066 SNPs were retained for GWAS (McCouch et al. 2016). After assessing the population structure (Zheng et al. 2012) of RDP1, GWAS analysis was performed in the R package rrBLUP (Endelman 2011) using a single marker linear mixed model:


y=1μ+Xβ+sα+Zg+ε

Here, *y* represents the vector of observations, *μ* represents the overall mean, *X* represents the design matrix for fixed effects, *β* represents the vector with principal components for population structure, *s* is the vector of gene content (0, 2) at a particular SNP locus, *α* is the SNP effect, *Z* is the design matrix for random effects, and ε is the vector of residuals, respectively. $g\;∼N(0.G\sigma_{g\;}^{2\;})$ is the vector of random effects that account for relatedness, where *G* is the genomic relationship matrix of accessions and $\sigma_{g\;}^{2\;}$ is the genetic variance.

Manhattan and *Q*–*Q* plots were generated using the qqman package in R (Turner 2014). Genome-wide significant SNP markers were obtained using the suggested threshold level of *P* < 3.3 × 10^−6^ or −log_10_(*P*) > 5.4 (Bai et al. 2016). Additionally, *R*^2^ values representing phenotypic variance contribution of each marker (or SNP) to the total variance and SNP heritability estimates were calculated using the GAPIT (Pérez and De Los Campos 2014) and the rrBLUP R (Endelman 2011) packages, respectively. We analyzed the LD between SNPs and SNPs within a LD block were categorized to be in a one QTL as given in Supplementary Table S1. The R package SNPRelate was used to perform LD and haplotype analysis (Zheng et al. 2012). Further, we scanned the 40 kb upstream and downstream of each SNP to get the nearby candidate genes. We used literature search to report to genes and QTLs previously known to control grain weight in rice.

### Field HNT stress experiment

We used multiple mobile high tunnel tents fitted with sensors and heating systems at a field experimental station in Harrisburg, AR. For this study, we filtered the *tropical japonica* subpopulation of RDP1 based on flowering time and selected 39 accessions with flowering times between 90 and 100 d. We selected this flowering time to maximize the number of accessions for HNT treatment. We obtained grain weight data from 29 HGA and 10 LGA allelic groups. These accessions were grown in each tent arranged in a randomized block design. For each accession, 16 seeds per plot (with a 7 cm spacing) were sowed using direct seeding. When 50% of plants reached flowering, 2 tents (each containing 1 plot per accession) were maintained at ambient conditions and 2 were exposed to HNT. The heat tents were operated to expose rice to HNT stress of ∼4 °C above the nighttime ambient (control) temperature. HNT stress was maintained for 2 wk, coinciding with flowering to grain filling stages of these accessions. The plots remained fully open during daytime. At the physiological maturity stage, 12 rice plants from each plot and SGW data were collected. ANOVA followed by LSD test was used in statistical analysis.

### Generation of LOGL1-mutants

To generate CR-based mutants (referred to as *logl1*), 2 single-guide (sg) RNAs targeting *LOGL1* exons were designed using CRISPR-P 2.0 (http://crispr.hzau.edu.cn/CRISPR/) (Lei et al. 2014). Two independent destination constructs, each containing a sg RNA (sg1 and sg2), were generated following a modified Gateway cloning method, as described in Lowder et al. (2015). For this, 2 sg sequences (sg1 and sg2) designed to target 2 different sites of *LOGL1* (Supplementary Table S11) were restriction cloned separately in pY1C (using Esp3I/BsmBI site) to get entry clones. The entry clones were recombined (using LR clonase) with pANIC6B (containing β-glucuronidase sequence) and pY7 (containing Cas9 sequence) and resulting destination clones were used for rice calli transformation, as described previously (Paul et al. 2020a). T1 plants lacking Cas9 (confirmed using negative β-glucuronidase assay) were screened for the presence of mutations using Sanger sequencing. The resulting 3 homozygous *logl1*-mutants independently targeting 2 different regions (*logl1*#1, *logl1*#2, and *logl1*#3) of *LOGL1* contained a 1 bp deletion for *logl1*#1, a 1 bp insertion for *logl1*#2, and a 41 bp deletion for *logl1*#3.

For creating OE lines, the rice *LOGL1* coding region was amplified from 2 DAF grain (Kitaake) cDNA using specific primers (Supplementary Table S11). The amplicon was cloned into pENTR/D-TOPO (Invitrogen). The entry construct was recombined with the destination vector pANIC6B containing maize ubiquitin 1 promoter. The final destination construct was used to transform rice calli (Kitaake). Homozygous *logl1-*mutant and OE plants from T2 were used for phenotypic evaluation.

### Genomic DNA and RNA extraction and RT-qPCR assay

For *logl1-*mutant screening, T1-*logl1* plants were screened for the absence of Cas9 using the GUS screening assay. Plants that lacked GUS staining (no blue) were then screened for the presence of mutations by Sanger sequencing. Genomic DNA isolated from T1 seedling leaves was used in polymerase chain reactions using primers flanking the sgRNA sites (Supplementary Table S11). The resulting amplicon was genotyped using Sanger sequencing, using primers given in Supplementary Table S11. The resulting sequencing reads were aligned with the Kitaake sequence to decipher the mutations. For expression analysis, developing grains without husk at 2 DAF were collected from mutants and WT. RNA extraction and reverse transcription reaction to obtain cDNA followed by quantitative real-time PCR (qPCR) analysis were performed as described previously (Sandhu et al. 2021). The gene-specific primers used in qPCR are specified Supplementary Table S11.

### *LOGL1-*mutant phenotyping

For accessing the HNT effect, 18 plants from WT, *logl1*, and OE were grown under a controlled greenhouse diurnal setting with a temperature of 28/23 ± 1.5 °C, light/dark 16/8 h and relative humidity of 55% to 60%. During peak flowering, 10 to 20 open florets per plant were marked and, at 1 DAF of marked florets, half of the plants were retained in control (28/23 ± 2 °C) and remainder moved to HNT greenhouse (30/28 ± 1.5 °C, light/dark 16/8 h). HNT treatment was maintained throughout grain development (Supplementary Fig. S8A). SGWs were obtained as described above for the RDP1 experiment. For grain area, length and width analysis, dehulled grains were scanned using an Epson Expression 12 000 XL scanner (resolution 600 dpi) and measurements were collected using the SeedExtractor app in MATLAB (Zhu et al. 2021). Grain thickness was measured using a Vernier caliper. For yield parameters of mutants and WT, all dehulled clean grains from each plant were used to calculate total grain weight and grain number per plant. The grain hormones were measured as described previously (Sandhu et al. 2021). The *t*-test was used in statistical analysis.

### RNA sequencing, GO term, and diurnal dataset

For transcriptome analysis of developing grains, actively flowering florets were marked at 1 DAF, and plants were either kept in the control greenhouse or moved to an HNT greenhouse. Developing grains (without husk) at 2 DAF from *logl1#*2, OE#2, and WT were snap-frozen in liquid nitrogen. Total RNA extracted from these samples was used to generate an RNAseq dataset, which was processed as described previously (Sandhu et al. 2021). Significantly (*P* adjusted < 0.1) differentially expressed upregulated [log_2_(foldchange) > 0.5] or downregulated [log_2_(foldchange) < −0.5] genes obtained from different pairwise comparisons were subjected to GO term analysis in PlantRegMap (Tian et al. 2020). The top biological terms enriched in datasets were plotted using the “ggplot2” package in R (Wickham 2016; R Core Team 2019). Rice diurnal expression for selected genes (Mockler et al. 2007) was plotted using the “heatmap2” function from the “gplots” package in R (Warnes et al. 2017). This dataset is generated from rice leaves collected every 4 h for 48 h (Filichkin et al. 2011).

### Accession numbers

Sequence data from this article can be found in the GenBank/EMBL data libraries under BioProject ID PRJNA855943. The gene ID for *LOGL1* is *LOC_OS01g51210*. The accession number (MSU Locus ID) of all other genes mentioned in the manuscript can be found in Supplementary Tables S4 and S6 to S11.

## Supplementary Material

kiae313_Supplementary_Data

## Data Availability

The RNA sequence data from this article has been submitted in GenBank/EMBL data libraries under BioProject ID PRJNA855943. Additional data is available in supplementary dataset.

## Supplementary data

The following materials are available in the online version of this article.

**Supplementary Figure S1.** Natural variation in response to high night temperature in RDP1.

**Supplementary Figure S2.** The *Q–Q* plots for SGW GWAS.

**Supplementary Figure S3.** Pattern of LD (*r*^2^) between HNT GWAS-associated SNPs on Chromosome 1.

**Supplementary Figure S4.** SGW of rice accessions in green house and field-based experiments.

**Supplementary Figure S5.** Impact of stacking favorable alleles for 3 major peaks on SGW under control (C) and HNT.

**Supplementary Figure S6.** Expression analysis for 14 protein-coding genes in the vicinity of *SGW1.*

**Supplementary Figure S7.** Allelic variation in expression of potential candidate genes near *SGW1* among HGA and LGA.

**Supplementary Figure S8.** *LOGL1* CR-based mutants (*logl1*) and OE.

**Supplementary Figure S9.** HNT experiment with WT, *logl1*-mutants and OE plants.

**Supplementary Figure S10.** Transcriptome of developing grains at 2 days after fertilization from WT, *logl1*#2 mutant, and over-expresser (OE#2) under control and HNT treatment.

**Supplementary Figure S11.** Amount of tZ and tZR in control (C) and HNT-treated grains and 2 and 4 days after fertilization (DAF).

**Supplementary Table S1.** List of RDP1 accessions used in the study, their HNT response and type of allele present in each accession for 3 major HNT SNPs.

**Supplementary Table S2.** Relationship between HNT response of SGW and panicle fertility. Number of sensitive, moderately resilient, and resilient accessions are given in the table.

**Supplementary Table S3.** The list of inferior and superior rice accessions identified based on their performance under HNT.

**Supplementary Table S4.** GWAS-based significant SNPs associated with SGW under control and HNT. Genes in the vicinity (within 40 kb of each SNP) of significant SNPs (location based on MSUv7.0) are enlisted. The 3 SNPs (*SGW1*, *sSGW6.1*, and *sSGW6.2*) used in favorable allele stacking analysis are highlighted.

**Supplementary Table S5.** LD between SNPs near SGW1.

**Supplementary Table S6.** List of 30 genes located in the vicinity of *SGW1*. Their developmental expression in the public dataset, GSE6893, and allelic variation in expression in RDP1 transcriptome (GSE98455) are reported. Here, NA: not applicable.

**Supplementary Table S7.** Statistical analysis comparing individual *logl1* and OE events with WT within control or HNT conditions.

**Supplementary Table S8.** List of 72 genes that were upregulated by HNT in WT and OE#2 but not in *logl1*#2.

**Supplementary Table S9.** GO terms for a set of 72 genes that were upregulated by HNT in WT and OE but not in *logl1*#2. GO term analysis was performed using PlantRegMap.

**Supplementary Table S10.** GO terms for genes that are differentially expressed between WT and *logl1* under control. GO term analysis was performed using PlantRegMap.

**Supplementary Table S11.** List of primers used in the study.

## Dive Curated Terms

The following phenotypic, genotypic, and functional terms are of significance to the work described in this paper:

THI1 Gramene: AT5G54770
THI1 Araport: AT5G54770

CK1 Gramene: AT4G26100
CK1 Araport: AT4G26100

NADPH CHEBI: CHEBI:16474

CCA1 Gramene: AT2G46830
CCA1 Araport: AT2G46830

THIC Gramene: AT2G29630
THIC Araport: AT2G29630

LHY Gramene: AT1G01060
LHY Araport: AT1G01060

TOC1 Gramene: AT5G61380
TOC1 Araport: AT5G61380

trans-zeatin CHEBI: CHEBI:16522

ROS Gramene: Reactive oxygen species
ROS Araport: Reactive oxygen species
