# Supplementary material for: Natural variation in LONELY GUY-Like 1 regulates rice grain weight under warmer night conditions
Source: Plant Physiol. 2024 May 31;196(1):164–80. doi: 10.1093/plphys/kiae313 (PMC11376391; doi:10.1093/plphys/kiae313)
Supplement: kiae313_Supplementary_Data [file kiae313_supplementary_data.zip › Supplementary Data_updated.pdf]

Supplementary Data

Supplementary Figure S1

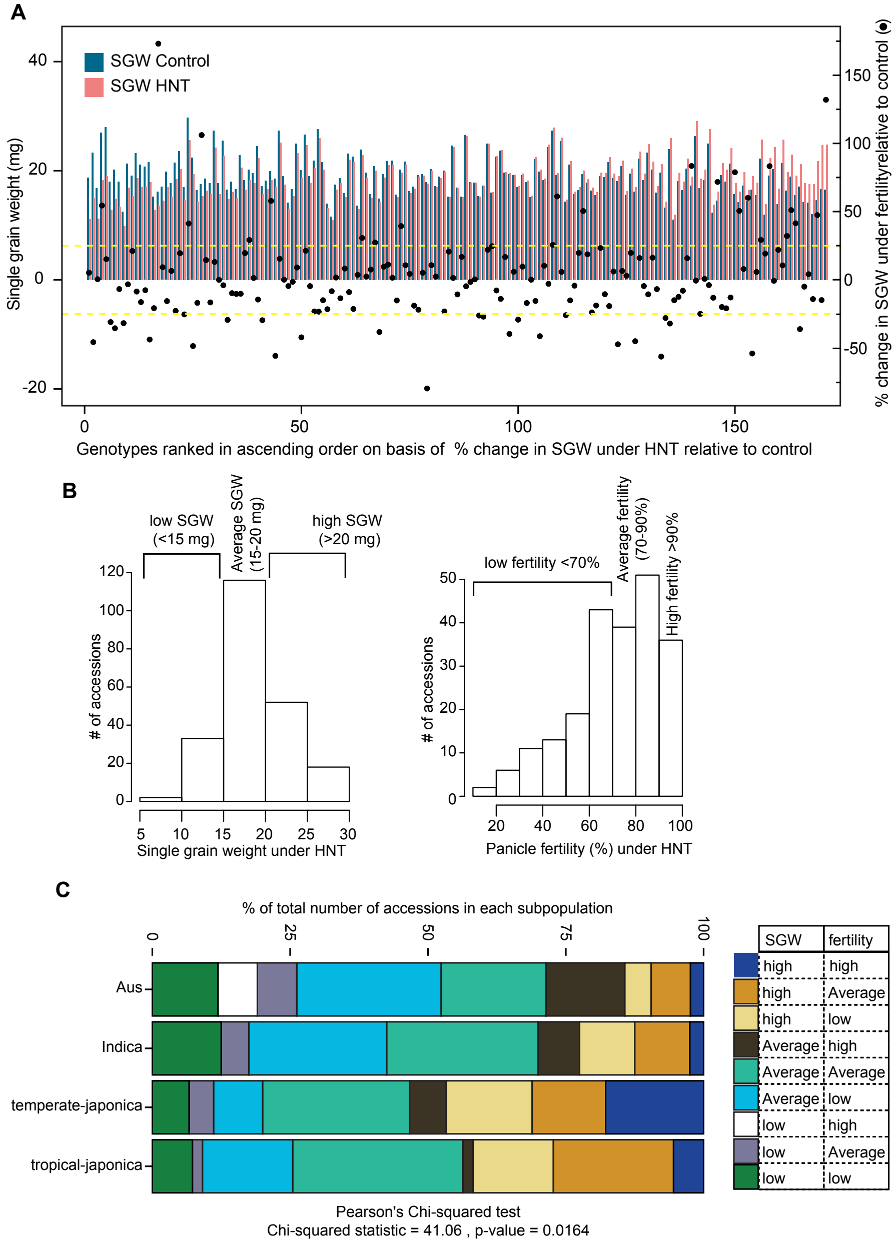

Supplementary Figure S1. Natural variation in response to high night temperature in rice diversity panel 1 (RDP1). (A) Extension of Figure 1A. Black dots represents natural variation % change in fertility ($\frac{HNT-control}{control} * 100$) in response to high night-time temperature (HNT) for rice diversity panel 1 (RDP1). Dotted yellow line represents 5% cutoff for percentage change in fertility of HNT-treated plants compared to control (on right Y-axis, represented by black dots). The bars represent single grain weight (SGW, bars) under control (blue) and HNT (red) conditions, and accessions are arranged in ascending order (left to right) based increased HNT sensitivity of SGW. **(B)** Categorization of RDP1 accessions based on distribution of single grain weight (SGW) and panicle fertility under high night-time temperature (HNT) treatments **(C)** Percent of total number of accessions within a subpopulation that belong to different categories that were determined based combined performance their SGW and fertility.

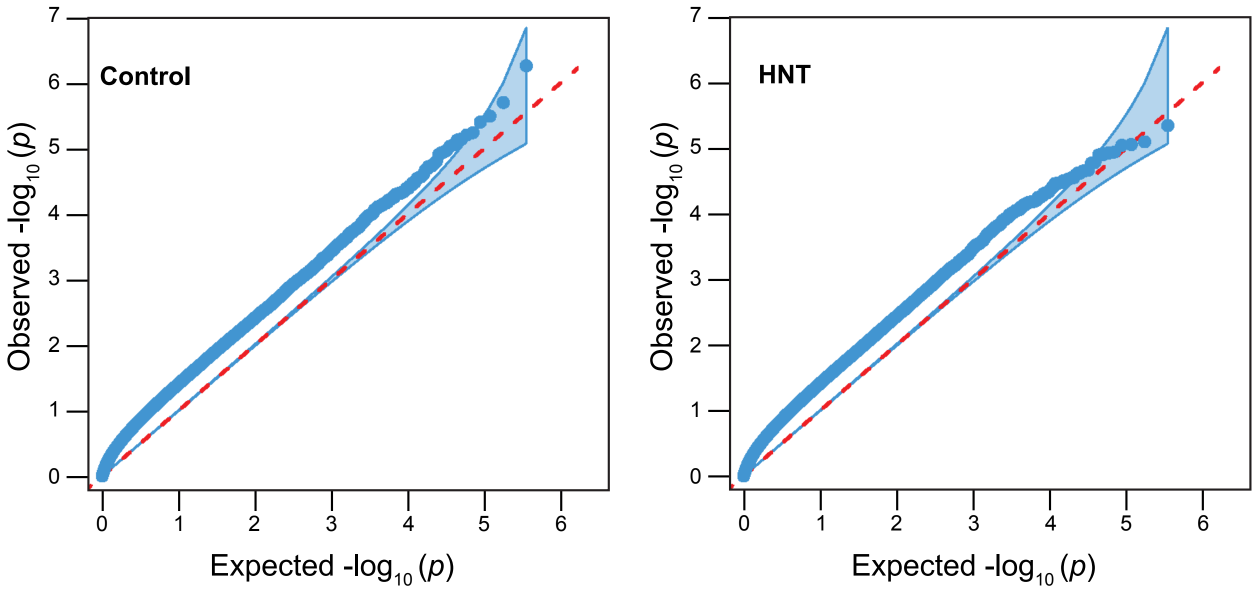

Supplementary Figure S2. The Quantile-Quantile (QQ) plots for single grain weight GWAS. The QQ plots of observed -log$_{10}$ (p) values against the expected probability of -log$_{10}$ (p) for single grain weight GWAS results using the linear mixed model under control and high night-time temperature (HNT) treatments. The light blue circles correspond to the observed -log$_{10}$ (p) values of markers derived from the mixed model GWAS. The red line indicates the expected -log$_{10}$ (p) values distribution under the assumption (null hypothesis) that the p-values follow a uniform distribution. The solid blue line filled with blue color shows the 95% confidence interval for the QQ-plot under the null hypothesis of no association between the SNP and the trait.

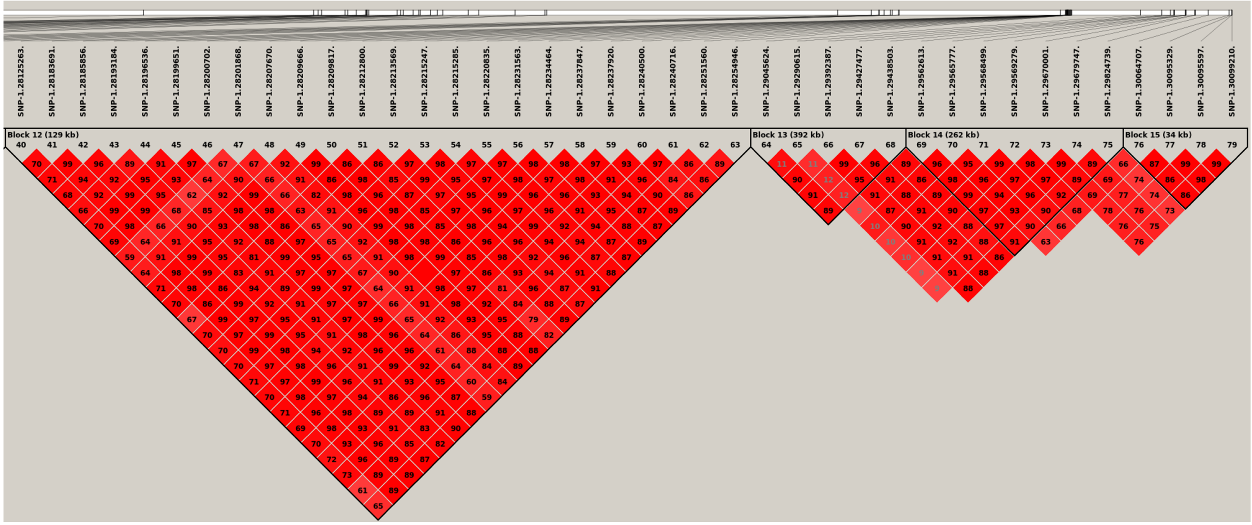

Supplementary Figure S3. Pattern of linkage disequilibrium (LD, r^2) between HNT GWAS-associated SNPs on chromosome 1 for the four haplotype blocks. The names of SNPs are given on the vertical axis in gray color and are positioned based on the physical distance (kb). The text represents the coefficient of LD (r^2) between the pairwise SNP combinations. The r^2 ranges from 0 to 1, where 0 means no LD between markers and 1 represents complete LD between the markers. The red squares with black color indicate $r^2 > 0.80$, and red squares with gray color indicate $r^2 < .50$. The solid block triangle represented by the black color represents the haplotype blocks of different sizes

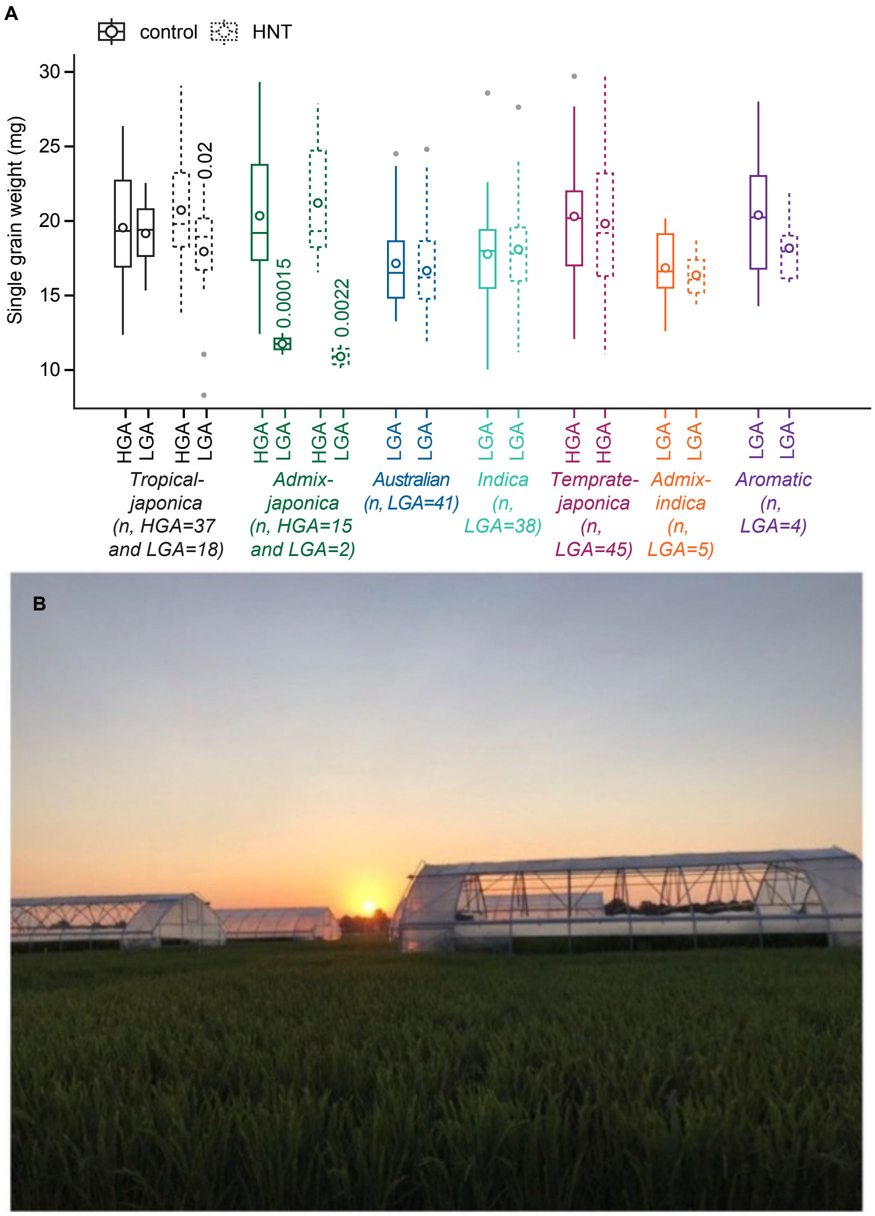

Supplementary Figure S4. Single Grain Weight of rice accessions in green house and field-based experiments. (A) Boxplot showing allelic effect of *Single Grain Weight 1* (*SGW1*) locus on single grain weight (SGW) in different subpopulations of the rice diversity panel 1 (RDP1) under control and high night-time temperature (HNT). *p*-Values (indicated by text) represent comparisons (t-test) between allelic groups within a treatment (control or HNT). In box plot, center line, median; box limits, upper and lower quartiles; whiskers, 1.5x interquartile range; grey points, outliers and unfilled circle inside box, mean. Heavy-grain accessions (HGA) and light-grain accessions (LGA) represent two allelic groups at *SGW1*. The n indicates number of accessions in LGA and HGA group within a subpopulation **(B)** Overall view of four high tunnel greenhouses and plot layout. When rice accessions reached flowering two back greenhouses (each containing one plot per accession) were maintained at ambient (control) conditions, while the front two were heated 4°C above ambient conditions to impose high night temperature (HNT) stress. The roofs, sidewalls, and end walls were rolled up during daytime (6 am to 7 pm) for normal plant growth.

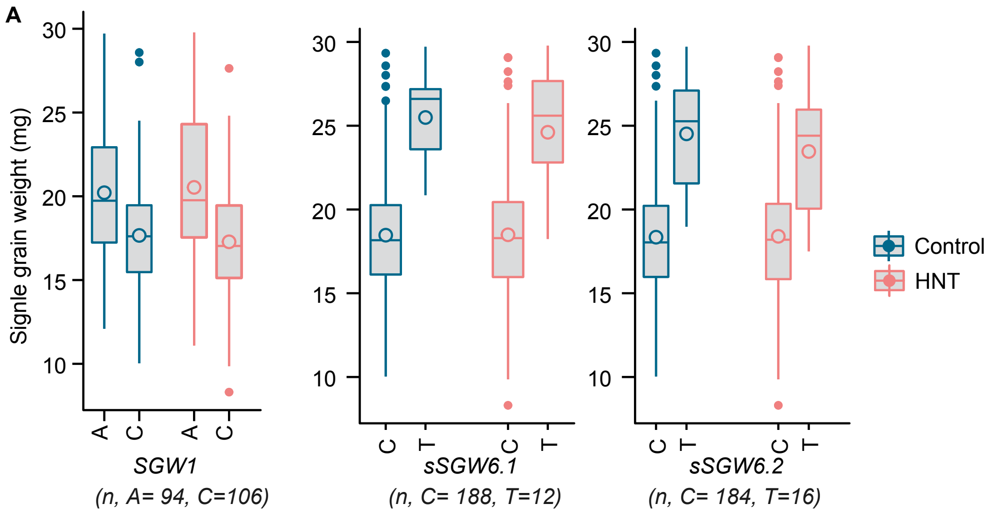

B

	SNP name	*SGW1*	*sSGW6.1*	*sSGW6.2*			
	SNP ID	SNP-1.29438503	SNP-6.17579336	SNP-6.20150777			
	Favourable Allele	A	T	T	Number of favourable alleles	Treatment	SGW
Haplotypes	Comb1	A	T	T	3	C	25.81[c]
	Comb1	A	T	T	3	HNT	24.52[bc]
	Comb2	A	C	T	2	C	22.48[abc]
	Comb2	A	C	T	2	HNT	23.07[b]
	Comb3	A	C	C	1	C	19.36[a]
	Comb3	A	C	C	1	HNT	19.75[a]
	Comb4	C	C	C	0	C	17.70[d]
	Comb4	C	C	C	0	HNT	17.31[d]

Supplementary Figure S5. Impact of stacking favorable alleles for three major peaks on single grain weight (SGW) under control (C) and high night-time temperature (HNT). (A) Boxplots showing allelic effect of three major loci, *SGW1 (LOGL1* intron SNP*), sSGW6.1,* and *sSGW6.2* on SGW. In box plot, center line, median; box limits, upper and lower quartiles; whiskers, 1.5x interquartile range; filled circles, outliers and unfilled circle inside box, mean; n, sample size. **(B)** Four combination groups (comb1, comb2, comb3 and comb4) obtained from allelic combination of three major loci (SNPs). For each of comb group, the type of alleles and number of favorable alleles are presented as table. SGW (means) with same significance letter are not significantly different from each other (t-test).

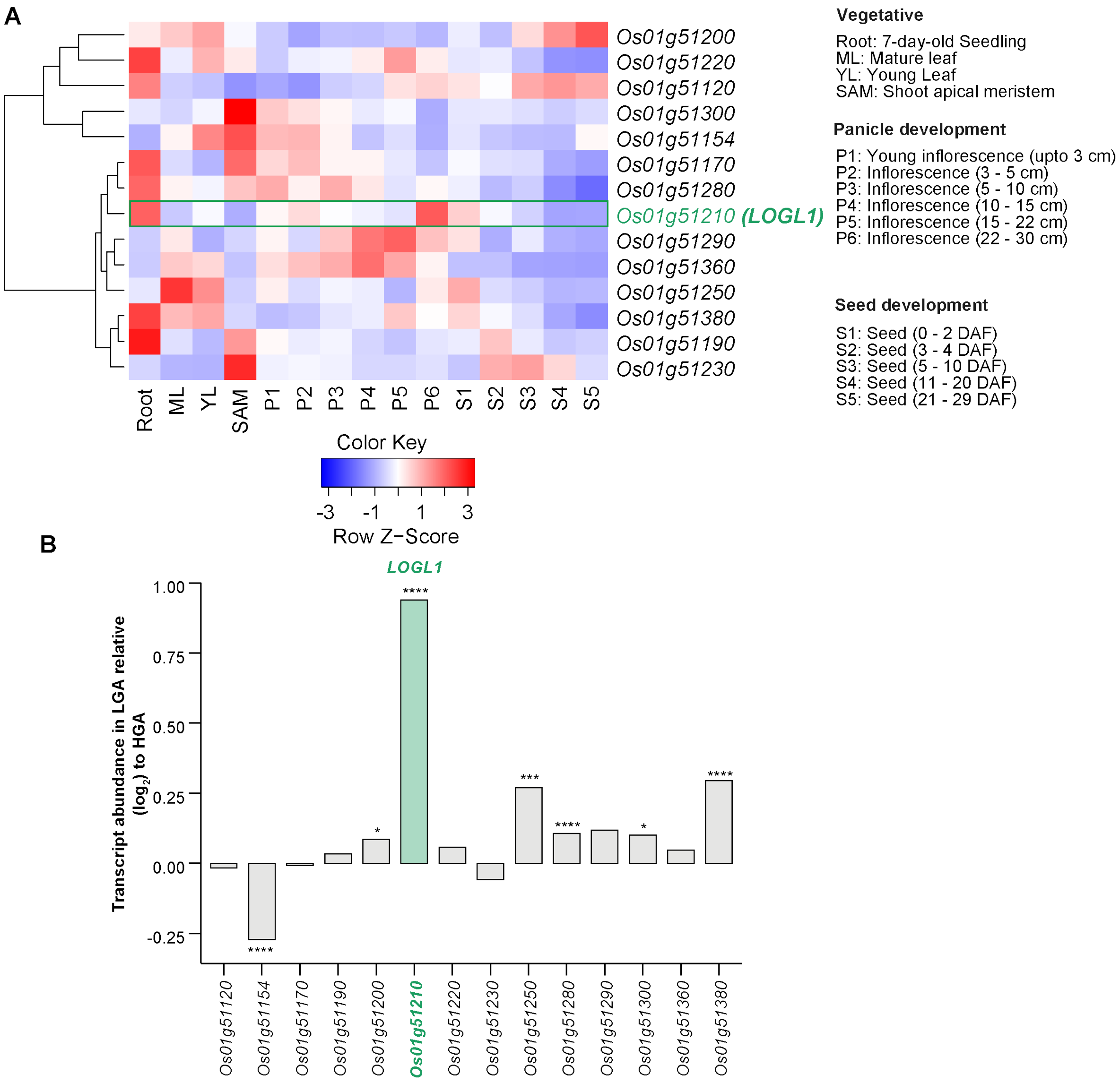

Supplementary Figure S6. Expression analysis for fourteen protein-coding genes in the vicinity of *SGW1*. These fourteen genes were selected from thirty genes within 100kb of *SGW1*. Remaining sixteen genes with no expression in grains were excluded from this analysis. **(A)** Expression of fourteen genes in different rice developmental stages and tissues (GSE6893[1]). *LOGL1* (highlighted in green) has preferentially higher expression in reproductive tissues (inflorescence and young seeds) than other developmental stages. **(B)** Fourteen genes were evaluated for transcript level allelic variations at *SGW1* locus using a public dataset (GSE98455[2]) for accessions from rice diversity panel 1. We used DESeq2 to obtain differentially expressed genes between light-grain accessions (LGA) and heavy-grain accessions (HGA) allelic groups. *LOGL1* (green) showed about ~2-fold higher transcript abundance in LGA than HGA allelic group accessions. Difference in transcript abundance for other genes between LGA and HGA were non-significant (p-adjusted cut-off = 0.1, $\log_2$ Fold-change cut-off = |0.5|). Here, (*, p-adjusted < 0.1; **, p-adjusted < 0.01; ***, p-adjusted < 0.001; ****, p-adjusted < 0.0001).

1. Jain, M. *et al*. F-box proteins in rice. Genome-wide analysis, classification, temporal and spatial gene expression during panicle and seed development, and regulation by light and abiotic stress. *Plant Physiol*. **143**, 1467–1483 (2007).

2. Campbell, M. T. *et al*. Characterization of the transcriptional divergence between the subspecies of cultivated rice (Oryza sativa). *BMC Genomics* **21**, (2020).

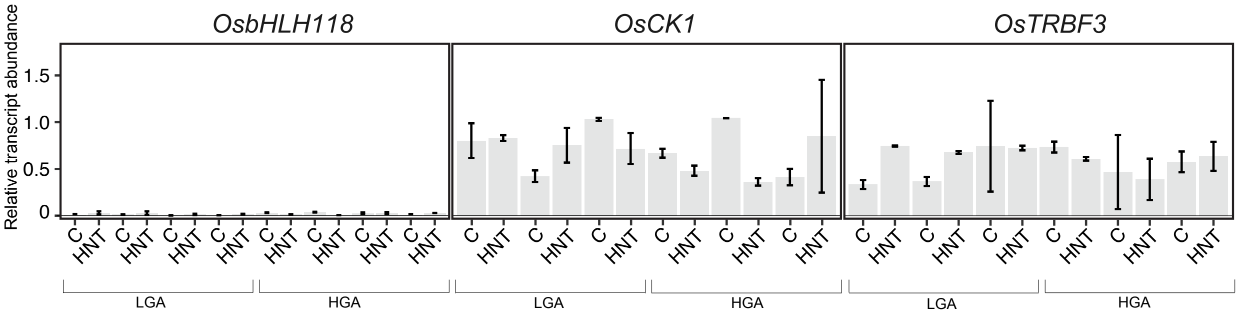

Supplementary Figure S7. Allelic variation in expression of potential candiate genes near *SGW1* among heavy grain accessions (HGA) and light grain accesions (LGA). The transcript abundace of *OsbHLH118*, *OsCK1* and *OsTRBF3* in grains at 2 DAF under control and high night temperature (HNT) was quantified using real-time quantitative PCR ($2^{-\Delta\Delta CT}$ method). The error bars are SD and n = 2 biological replicates with more than 25 seeds per replicate obtained from 3-4 plants. The four accesions from LGA are NSFTV-24, NSFTV-251, NSFTV-258, NSFTV-337 and from HGA are NSFTV-56, NSFTV-73, NSFTV-113 and NSFTV-303.

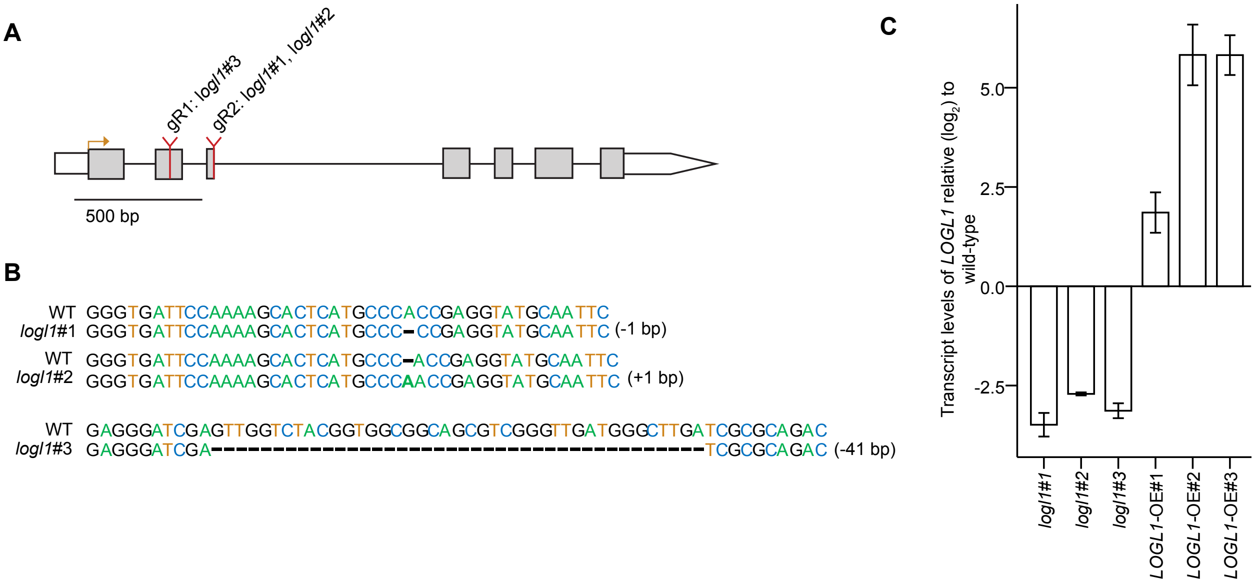

Supplementary Figure S8. ***LOGL1*** **CRISPR-Cas9 based mutants (*logl1*) and overexpression (OE).** **(A)** *LOGL1* gene structure with positions (red) of single guide RNA 1 (Sg1) and Sg2. The corresponding *logl1*-mutant alleles obtained from each of guide RNA are indicated with black text. The golden arrow indicates the start codon, ATG. **(B)** Sequence analysis of homozygous *logl1*-mutant alleles. Text in parenthesis represents type of mutation. Three homozygous *logl1*-mutants independently targeting two different regions (sg2, #1, and #2; sg1, #3) of *LOGL1* had 1 bp deletion for #1, 1 bp insertion for #2 and 41 bp deletion for #3. **(C)** Relative transcript abundance (mean ± SD) of *LOGL1* in two days after fertilization old grains under control conditions. and n = 2 biological replicates with more than 25 seeds per replicate obtained from 3-4 plants.

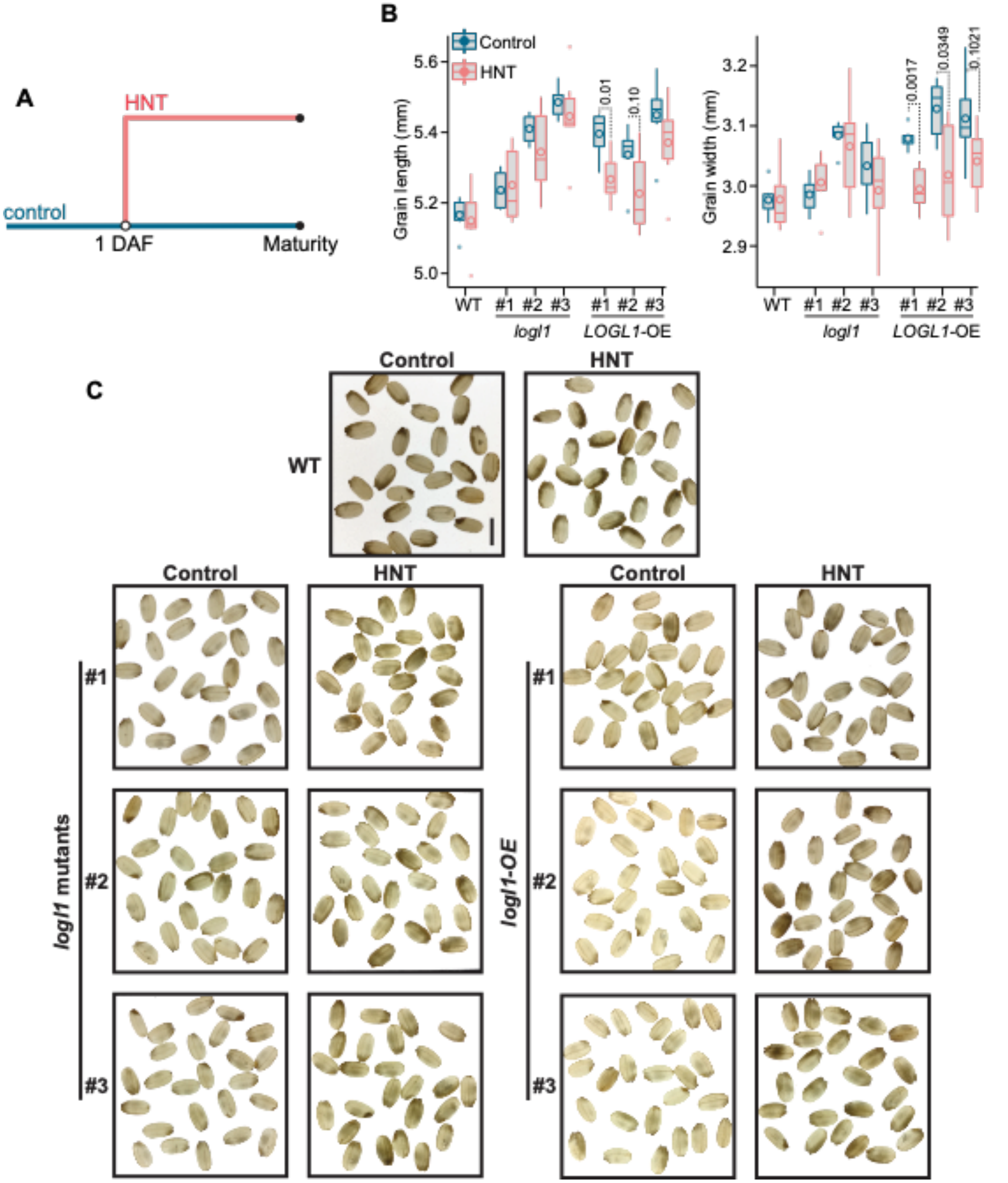

Supplementary Figure S9. Hight night temperature experiment with experiment with wild type (WT), *logl1*-mutants and OE plants. **(A)** Schematic representation of the experiment. **(A)** Schematic representation of high night-time temperature (HNT) experiment. Florets were marked at time of flowering, and at 1 d after flowering (DAF). Wild-type (WT), over-expression (OE) and *logl1*-mutant plants were exposed to either control (C) or high night temperature (HNT) treatment until physiological maturity. **(B)** Grain size under control and HNT. Box plot represents range, median and mean (filled circle inside boxplot) for six plants. Significant difference (t-test) between C and HNT within each genotype is indicated by *p*-values. **(C)** Light-box images of control and HNT treated grains of WT, OE, and mutant grains. The seeds are spread on a lightbox with underneath light source. Scale bar, 5mm.

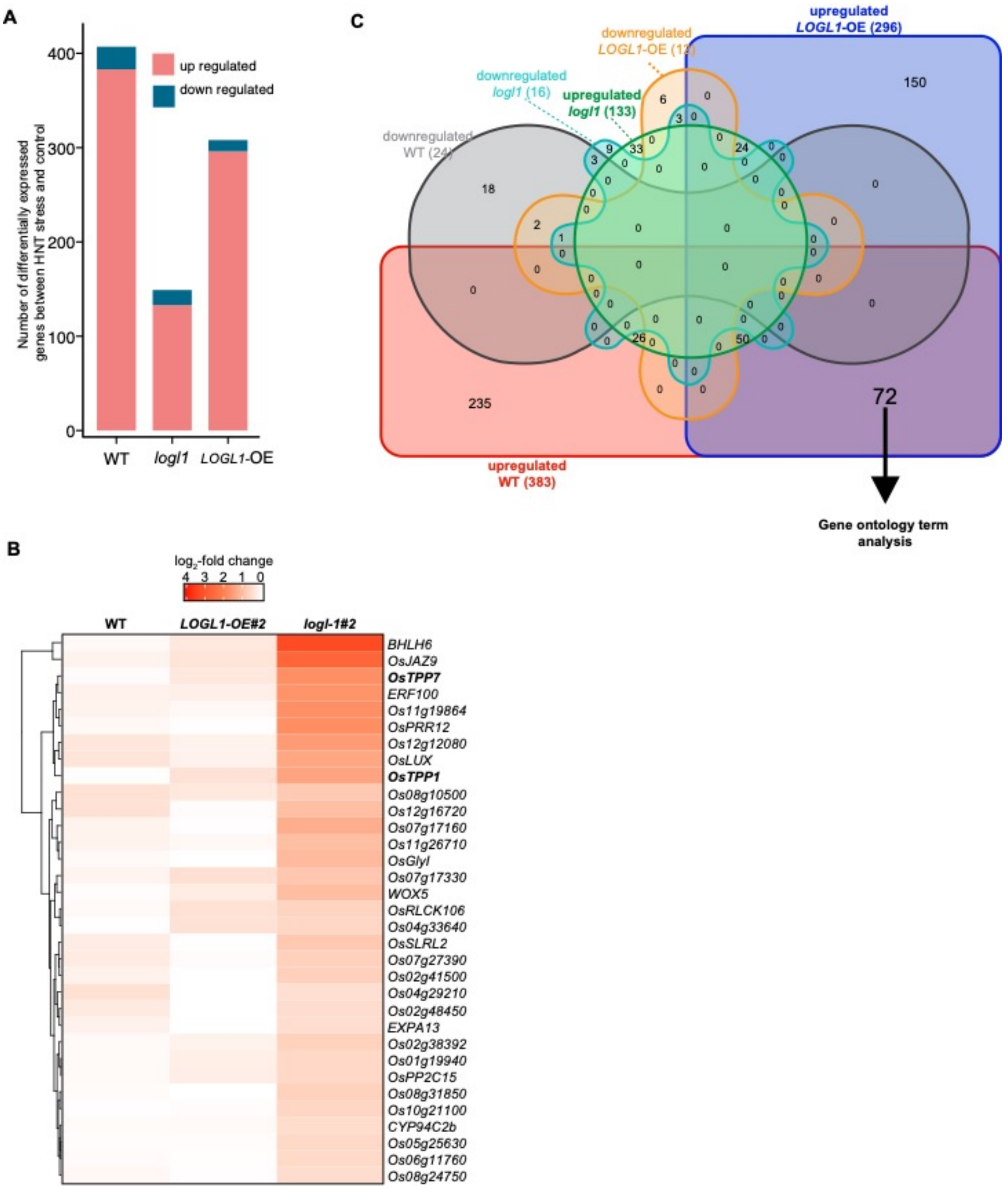

Supplementary Figure S10. Transcriptome of developing grains at 2 days after flowering (DAF) from wild-type (WT), *logl1*#2 mutant and over-expresser (OE#2) under control and high nighttime temperature (HNT) treatment. **(A)** Number of differentially expressed genes (DEGs) between HNT and control for WT, *logl1* and OE genotypes. **(B)** Heat map representing relative (HNT compared to respective controls) expression of genes that are significantly upregulated (adjusted-$p < 0.1$, $\log_2$-fold change > 0.5) by HNT in *logl1*-mutant but were not sensitive to HNT for WT and OE. **(C)** A Venn diagram showing the overlap of HNT-regulated genes among different comparisons. Differentially up- or down-regulated genes between HNT and control for WT, *logl1* and OE were analyzed to obtain overlapping number. The 72 genes (black arrow) that were upregulated by HNT for WT and OE but not for *logl1* were further subjected to Gene Ontology (GO) term analysis.

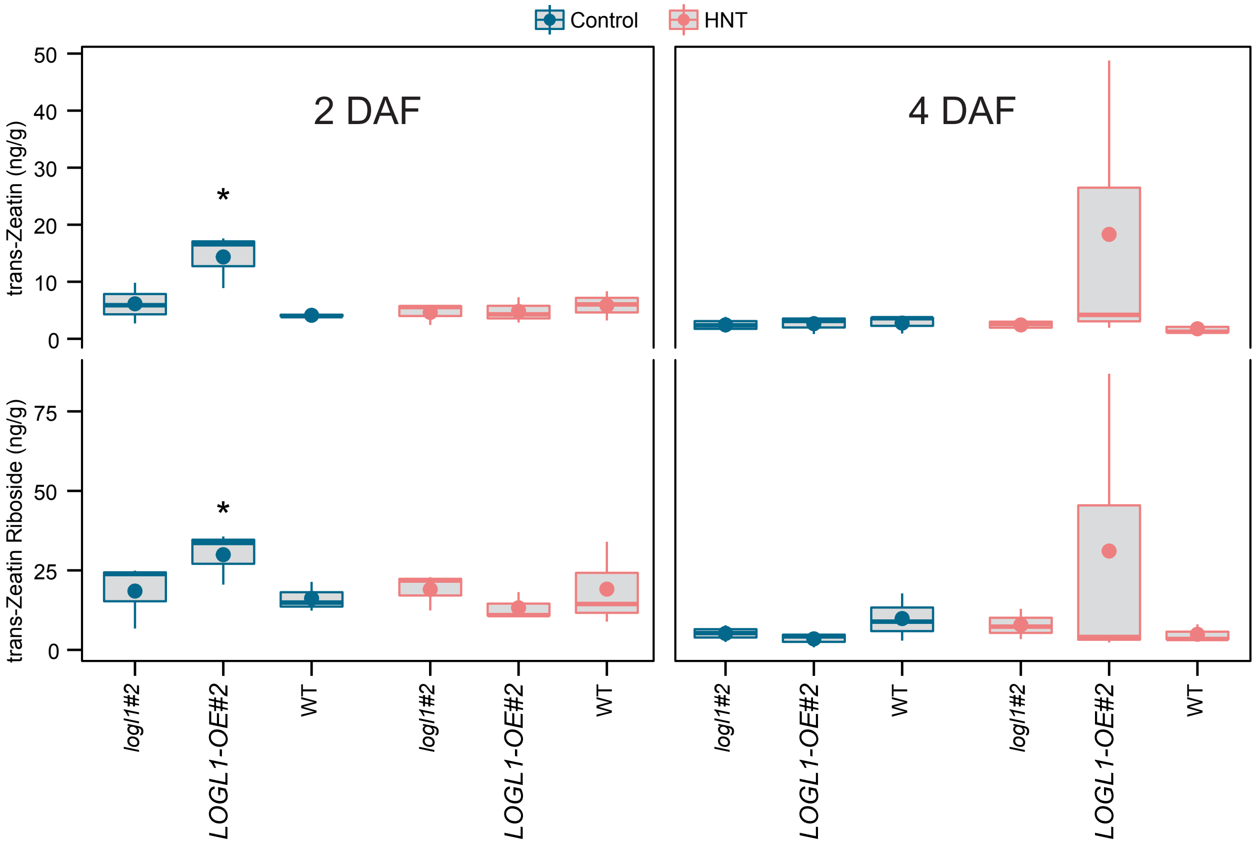

Supplementary Figure S11. Amount of trans-Zeatin and trans-Zeatin Riboside in control (C) and HNT treated grains and 2 and 4 days after fertilization (DAF). Here, * $p < 0.1$ and t-test was used to compare *LOGL1-OE#2* and *logl1#2* to WT. n, 2 biological replicates with more than 25 seeds per replicate obtained from 3-4 plants.

Supplementary Table S2. Relationship between high nighttime temperature (HNT) response of single grain weight (SGW) and panicle fertility. Number of sensitive, moderately resilient and resilient accessions are given in the table.

		Fertility		
SGW		Sensitive<br>more than 5% reduction	Resilient<br>% change > -5%	**Total**
Sensitive	more than 5% reduction	32	30	62
Moderately resilient	increase or decrease is <5%	27	39	66
Resilient	more than 5% increase	20	29	49
